# Prevalence of plant beneficial and human pathogenic bacteria isolated from salad vegetables in India

**DOI:** 10.1186/s12866-017-0974-x

**Published:** 2017-03-14

**Authors:** Angamuthu Nithya, Subramanian Babu

**Affiliations:** School of Bio Sciences and Technology, VIT University, Vellore, 632014 India

**Keywords:** Bacteriome, Endophyte, Human pathogenic, Plant beneficial, Salad, Vegetables

## Abstract

**Background:**

The study aimed at enumerating, identifying and categorizing the endophytic cultivable bacterial community in selected salad vegetables (carrot, cucumber, tomato and onion). Vegetable samples were collected from markets of two vegetable hot spot growing areas, during two different crop harvest seasons. Crude and diluted vegetable extracts were plated and the population of endophytic bacteria was assessed based on morphologically distinguishable colonies. The bacterial isolates were identified by growth in selective media, biochemical tests and 16S rRNA gene sequencing.

**Results:**

The endophytic population was found to be comparably higher in cucumber and tomato in both of the sampling locations, whereas lower in carrot and onion. Bacterial isolates belonged to 5 classes covering 46 distinct species belonging to 19 genera. Human opportunistic pathogens were predominant in carrot and onion, whereas plant beneficial bacteria dominated in cucumber and tomato. Out of the 104 isolates, 16.25% are human pathogens and 26.5% are human opportunistic pathogens.

**Conclusions:**

Existence of a high population of plant beneficial bacteria was found to have suppressed the population of plant and human pathogens. There is a greater potential to study the native endophytic plant beneficial bacteria for developing them as biocontrol agents against human pathogens that are harboured by plants.

**Electronic supplementary material:**

The online version of this article (doi:10.1186/s12866-017-0974-x) contains supplementary material, which is available to authorized users.

## Background

Fresh vegetables are considered as the essential components of healthy diet of people and the consumption of vegetables in the form of salads has increased in many parts of the world, including India. In contrast to the potential health benefits of fresh vegetables, a concern about the safety and the quality of vegetables has also raised due to outbreaks of infectious diseases reported from by Center for Disease Control and Prevention (CDC), US Food and Drug Administration (FDA), World Health Organization (WHO) and Center for Science in the Public Interest (CSPI). These changes are mainly due to change in the ecology of human pathogens to persist in non-host environments.

Since the contamination of salad vegetables with human pathogenic bacteria has reached concerning proportions in recent years, which has been evidenced by reports of various public health agencies through enhanced epidemiological and surveillance techniques, the raw vegetables are undoubtedly the portable source of infectious microorganisms, which has been revealed by numerous outbreaks associated with the consumption of salad vegetables [1, 2].

In general, fresh vegetables are known to harbour large bacterial populations [3], which may be of plant endophytes, plant pathogenic and human pathogenic in nature. The most important features of plant host colonization is by the adaptation of pathogens to the host defence response, physiology, immunity, native microflora, physical barriers, mobility and temperature. The pathogenic or non-pathogenic bacteria have several points of opportunities to contaminate fresh vegetables from the field through the time of consumption [4]. However, the route cause for the contamination of these vegetables, survival rate of endophytic bacterial communities, survival rate of pathogens which may be for plant or/and human, their interaction strategies, survival mechanisms are still under exploration.

Since the fresh vegetables in the form of salads are consumed raw, the pathogens present in it lead to widespread disease outbreaks. The non-pathogenic microbes associated with plants as a commensal or pathogen may leads to allergies which is still undeterminable due to change in the interaction strategies of microbes with the endophytic bacterial community and the plant host [5]. There are several reported outbreaks related to salad vegetables from the past decades to the present. Recently, Listeriosis outbreak was reported by CDC on January 28, 2016 from the consumption of mixed salad vegetables; *E. coli* 0157 outbreak was reported by USA Today newsletter related to salad vegetables in costco chicken; multistate *Salmonella* outbreak was linked to cucumbers in 2015 and thus the outbreaks are expanding.

In order to step on to the control of these outbreaks, detail reports of the endophytic bacterial community of vegetables used for salads, survival rate of pathogens in non-host environment etc. have to be identified. The present study was undertaken to find the endophytic bacterial community of the most commonly used South Indian salad vegetables like carrot, cucumber onion and tomato sold in vegetable markets. The study was undertaken by collecting the vegetable samples from two different vegetable growing hot spots of Tamil Nadu, India. Further, the endophytes were classified based on the evolutionary relationship to identify the predominant endophytic taxonomical group in salad vegetables and further classified based on their specific known functions such as human pathogens, human commensals, plant pathogens, plant commensals and environmentally beneficial bacteria.

## Methods

### Sampling method and surface sanitization

Fresh, damage-free whole salad vegetable samples (carrot, cucumber, onion and tomato) were purchased from two different local markets in Tamil Nadu, India (Hosur and Salem) during April and October of 2015. In Tamil Nadu, these two places are situated in the North Western agro climatic zone, but they differ in their soil type. The vegetable samples were collected in sterile plastic bags and transported to the laboratory as soon as possible. Samples were stored at 4 °C and tested within 48 h. Carrot, cucumber and tomato samples were washed with sterile deionized water and the external surface was scrubbed with an alcoholic solution of iodine (2%), and allowed to air dry inside a laminar airflow cabinet. The outer layer of onion was peeled and washed with deionized water and the external surface was scrubbed with an alcoholic solution of iodine (2%). Sterilization efficacy was evaluated by cutting the scrubbed external surface with sterile scalpel blade and placing directly on the surface of nutrient agar medium. The method chosen for sterilization, treatment with alcoholic solution of 2% iodine and drying under UV light on each side of vegetables, proved to be effective in killing the surface associated bacteria. For every sample batch, two samples of each vegetable were randomly chosen following surface sterilization and placing the external surface on nutrient agar plate. After 24 h of incubation at 37 °C, none of the tested vegetables showed bacterial growth.

### Endophytic bacteriological analysis of salad vegetables

Twenty five grams of each salad vegetable sample was weighed aseptically and homogenized by blending in 225 mL of sterile buffered peptone water using commercial blender. One millilitre of each homogenate was mixed with 9 mL of sterile 1% buffered peptone water in a sterile test tube, labelled 1:10 (10^-1^) dilution and subsequent dilution was done in five other test tubes labelled 10^-2^, 10^-3^ and 10^-4^. The same procedure was repeated for each sample and the blender was cleaned carefully and disinfected in between each samples to prevent cross contamination. For each vegetable, 100 μL of undiluted (crude extract) and diluted (10^-2^, 10^-3^, 10^-4^) samples were plated separately on nutrient agar plates (HiMedia, Mumbai, India) by standard spread plate technique and incubated aerobically at 37 °C for 24 h. All the discrete colonies were counted and expressed as colony forming units per gram (CFU g^-1^) of vegetable samples. Plating was done in three replications and the colony count was averaged.

### Isolation and biochemical characterization of endophytic bacteria

Pure cultures were obtained by streaking isolated single colonies on nutrient agar plate by quadrant streak method and incubated at 37 °C for 24 h. Colonies were presumptively distinguished based on the colony morphology on nutrient agar and selective media including AIA (*Aeromonas* Isolation Agar), CA (Certemaid Agar), EMBA (Eosin Methylene Blue Agar), MSA (Mannitol Salt Agar), SSA (*Salmonella*—*Shigella* Agar) and further tested for Gram’s reaction, M (motility), I (Indole), MR (Methyl Red), VP (Voges – Proskauer), C (Citrate), C (Catalase), O (Oxidase) and N (Nitrate) and incubated at 37 °C for 24 h.

### Isolation of DNA from endophytic bacteria

Endophytic bacterial isolates from different salad vegetables were grown on nutrient agar plate and incubated overnight at 37 °C. A single colony was suspended in a micro-centrifuge tube containing 30 μl of sterile nuclease free water and heated at 94 °C for 10 min. The sample was cooled to room temperature and centrifuged at 10,000 rpm for 10 min and the supernatant was directly used as template DNA for PCR.

### Molecular identification of endophytes by 16S rRNA gene sequencing

Partial sequencing of the 16S rRNA gene was done by PCR amplification of endophytic bacterial isolates using universal primers. SBBUF (5’-AGAGTTTGATCATGGCTCAG-3’) and SBBUR (5’-TACGGCTACCTTGTTACGAC-3’) primers were used as forward and reverse primers, respectively. PCR amplification was carried out in a thermocycler (Eppendorf, Mastercycler, Hamburg, Germany). The reactions were routinely performed in 20 μl: 2 μl of 30 ng/μl endophytic bacterial DNA, 1 μl of 0.5 μM of each of the opposing amplification primers, 6 μl of nuclease free water and 10 μl of 1X PCR master mix (Ampliquon, Denmark) containing 1.5 mM MgCl_2_,Tris HCl pH 8.5, (NH_4_)_2_SO_4_, 0.2% Tween 20, 0.4 mM dNTPs, 0.2 U/μl Taq DNA polymerase, inert red dye and stabilizer. PCR amplification program for 16S rRNA primers includes an initial denaturation of template DNA at 94 °C for 10 min followed by 30 cycles with a denaturation step at 94 °C for 1 min, annealing at 49 °C for 1 min, and extension at 72 °C for 1 min, and final extension at 72 °C for 7 min. PCR amplified products were separated on 1% agarose gels containing ethidium bromide with molecular weight marker and visualized under a UV light. PCR products were purified and sequenced at Amnion Bioscience Pvt. Ltd. (Bengaluru, India) to determine the diversity and composition of the salad vegetables associated bacterial communities. Sequencing was performed with SBBUF and SBBUR primers using ABI 3730xl genetic analyser. The resulted nucleotide sequences were edited for any overlaps using Chromas Lite software.

### Sequence analysis and classification

To ascertain the phylogenetic affiliation of the endophytic bacterial isolates, partial nucleotide sequences were assigned bacterial taxonomic affiliations based on the closest match to sequences available in the GenBank database (https://blast.ncbi.nlm.nih.gov/Blast.cgi) using the Basic Local Alignment Search Tool (BLAST) search algorithm. Sequence comparisons were done by constructing a sequence data matrix of 16S rRNA genes of each endophytic bacterial isolate and further classified based on their nature of existence as human pathogen, human opportunistic pathogen, plant pathogen, plant beneficial, plant commensal and environmentally beneficial bacteria.

Phylogenetic trees were constructed based on neighbour-joining [6] and maximum-parsimony method using MEGA software, version 6 [7]. Bootstrap analyses were performed for 1000 replications. The nucleotide positions containing gaps and missed data were eliminated. The 16S rRNA gene sequences determined in the study was deposited in the GenBank sequence library and their accession numbers are indicated on the tree.

### Diversity indices of endophytic bacteria

The diversity indices of the endophytic bacterial isolates were calculated by using the PAST program version 2.10 [8].

## Results

The results of this study provide insight into the identification of dominant members of the endophytic bacterial communities on the common South Indian salad vegetables such as carrot, cucumber onion and tomato.

**Fig. 1:**
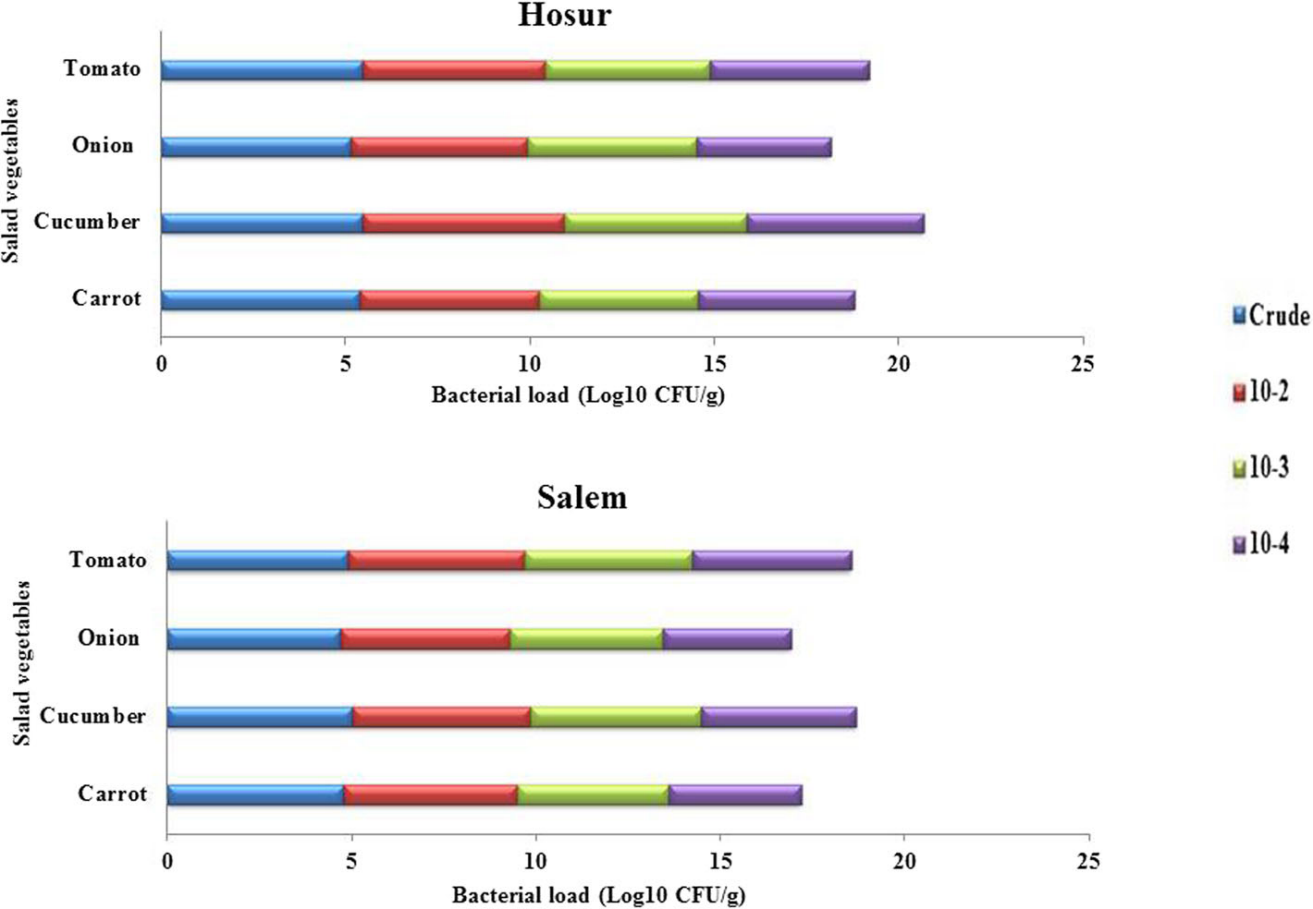
Endophytic bacterial population from internal tissues of salad vegetables

The endophytic population was found to be comparably higher in cucumber and tomato in both of the sampling locations, whereas lower in carrot and onion (Fig. 1). The per gram fresh weight population density of endophytic bacterial isolates in carrot ranged from 5.4 × 10^-1^ (crude extract) to 3.6 × 10^-4^ (10^-4^ dilution) CFU g^-1^, that of fresh cucumber ranged from 5.5 × 10^-1^ (crude extract) to 4.2 × 10^-4^ CFU g^-1^(10^-4^ dilution), fresh onion from 5.1 × 10^-1^ (crude extract) to 3.4 × 10^-4^ CFU g^-1^ (10^-4^ dilution) and of fresh tomato from 5.5 × 10^-1^ (crude extract) to 4.3 × 10^-4^ CFU g^-1^(10^-4^ dilution). The reduction in population density by dilution of vegetable extract was more in onion compared to other vegetables. A total of 104 endophytic bacterial isolates were isolated from four vegetables based on their distinguishable colony morphology (shape, size, colour and margin). The total number of morphologically distinguishable isolates obtained from crude and diluted extracts of tomato collected in two seasons is 23 (Hosur – 16; Salem – 7), cucumber is 31 (Hosur – 21; Salem – 10), carrot is 30 (Hosur – 24; Salem – 6) and onion is 20 (Hosur – 14; Salem – 6). In the case of carrot collected from both locations and tomato from Salem, all the isolates are either from crude extract or 10^-2^ dilution. The isolates were designated as SBAN (authors name), followed by location of sample (H-Hosur; S – Salem), common name of vegetable (CA or Ca – carrot; Cu – cucumber; O – onion; T – tomato) and the isolate number. Isolates obtained from crude extract and dilutions were numbered in sequential order. Since designation of some of the isolates were found to be overlapping after GenBank submission, they were labelled as 1a, 2a etc for the purpose of differentiation.

The endophytic bacterial isolates were further characterized by observing their growth and colony morphology on selective media. On AIA media, only one isolate showed growth (SBANHCu11) and it was from Hosur cucumber. On CA media, the isolates SBANHCa1, SBANHCA14, SBANHCu19, SBANHCu20, SBANHCu22, SBANHCu25, SBANCu25a, SBANSCu19, SBANSCu20, SBANSCu22, SBANHO5, SBANHO8 and SBANHO14 exhibited yellowish-green to blue colonies, which represented the presence of *Pseudomonas* spp. EMB agar screening revealed no isolates with green-metallic sheen, which represents the absence of *E. coli.* SBANHCA2, SBANHCa7a, SBANHCa10, SBANHCa11, SBANHCa14a, SBANSCu7, SBANHO2, SBANHO15, SBANSO90, SBANHT7, SBANHT9, SBANHT14 and SBANHT15 isolates showed yellowish colonies in MSA media, which indicated the presence of *Staphylococcus* spp. and on SS agar medium the isolates SBANHCA3, SBANHCa4 and SBANHT11 showed black colour colonies, representing the presence of *Salmonella* spp.

Further, biochemical characterization of endophytic bacterial isolates was done in which, about 57.7% (*n* = 60) of the isolates were identified as Gram positive and the remaining 42.3% (*n* = 44) of the isolates as Gram negative. The existence of rod shaped endophytic bacterial population were about 87.5% (*n* = 91) and the cocci were about 12.5% (*n* = 13). In motility test, 79.8% (*n* = 83) were found to be motile and 20.2% (*n* = 21) were non-motile. The isolates were also classified based on I, MR, VP, C, catalase, oxidase and nitrate test, and the results are shown in Additional file 1: Table S1(a), (b), (c) and (d).

Molecular characterization of all the 104 endophytic bacterial isolates was carried out by PCR amplification of the genomic DNA using universal bacterial primers for 16S rRNA gene. The expected size of 1.2 kb fragment for each of the 104 isolates was obtained and sequenced. The nucleotide sequences were searched for homology in the NCBI GenBank database using the BLASTn program. The results showed highest homology of > 97 to 100. Based on the 16S rRNA gene sequence identity, bacterial isolates were found belonging to 5 classes namely actinobacteria (11%), bacilli (43%), cocci (4%), betaproteobacteria (3%) and gammaproteobacteria (39%) with 46 distinct species belonging to 19 genera. The data also indicated the existence of more diverse endophytic bacterial communities in tomato and cucumber in both the sampling locations as compared to onion and carrot. Among the various endophytic bacterial populations, the class bacilli was found to be predominant with 21 diverse genera followed by gammaproteobacteria (14 genera), actinobacteria (8 genera), betaproteobacteria (2 genera) and cocci (1 genera). Actinobacteria was found to be distributed high in cucumber compared to other salad vegetables. Distribution of bacilli was high in tomato, whereas cocci were predominantly present in carrot. Betaproteobacteria and gammaproteobacteria were high in cucumber.

**Fig. 2:**
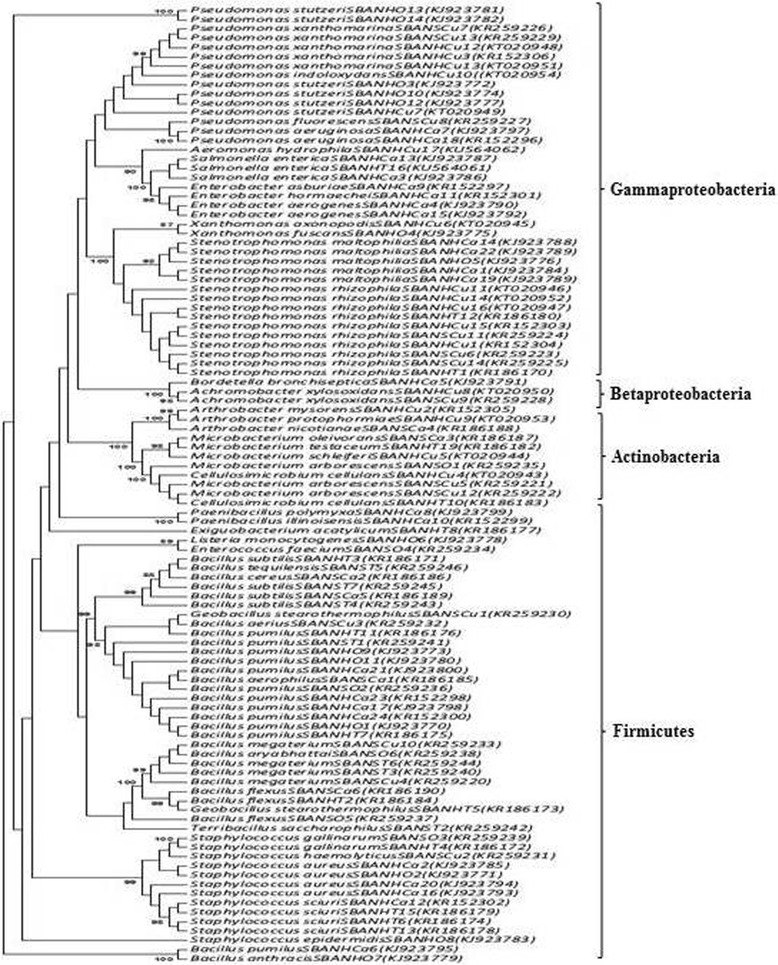
Phylogenetic tree of endophytic bacteria in salad vegetables based on 16S rRNA gene sequences

**Fig. 3:**
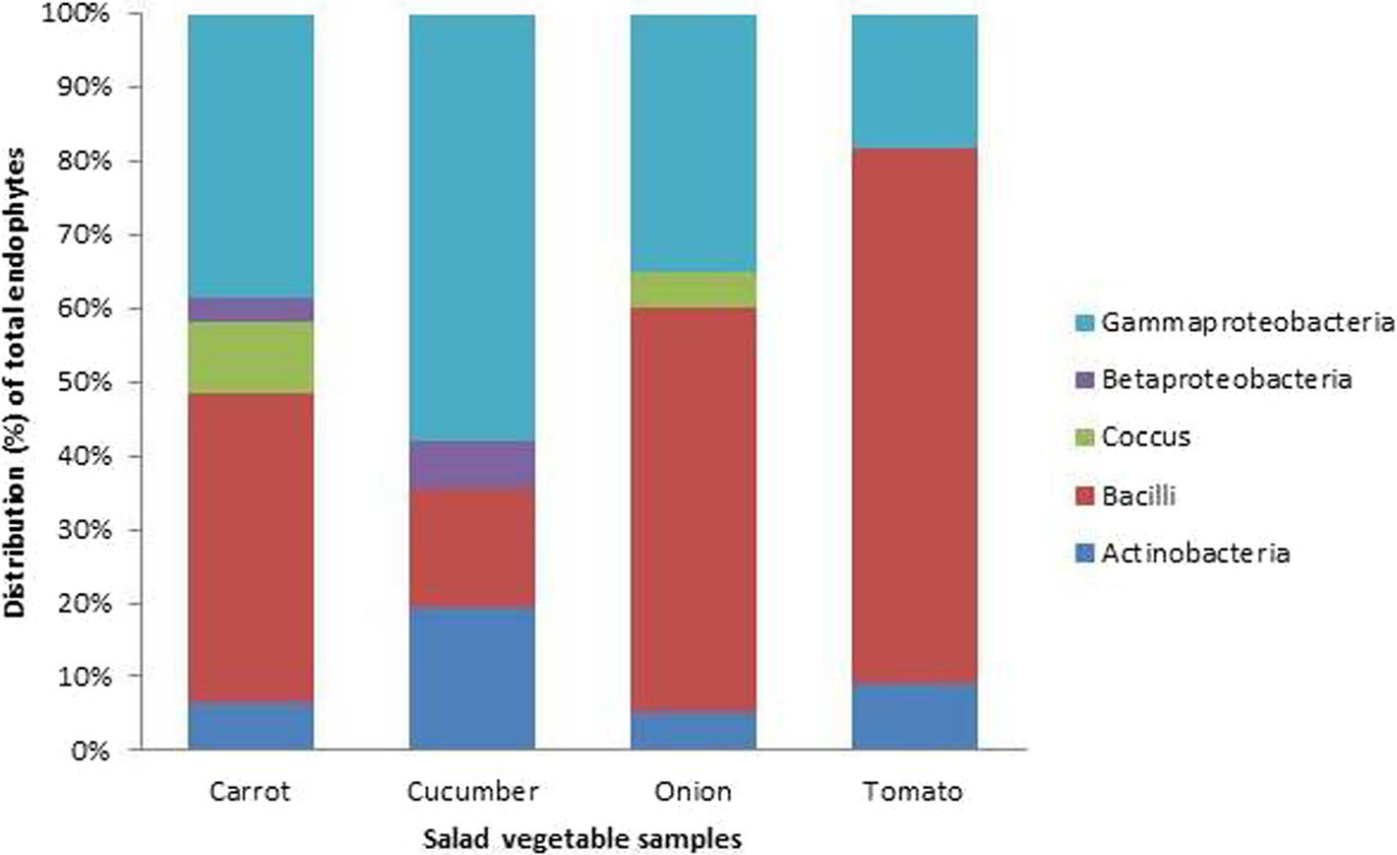
Taxonomical distribution of endophytic bacteria in salad vegetables

The taxonomical classification of endophytic bacterial isolates of salad vegetables observed in the study is presented in Table 1. Phylogenetic analysis was done by constructing the neighbour joining phylogenetic tree of all the 104 endophytic bacterial isolates to explore the evolutionary relationship, and the evolutionary distance was calculated using maximum composite likelihood method using MEGA software, version 6. Figure 2 represents the phylogenetic tree based on 16S rRNA sequences of the isolated bacteria. The overall class distribution of bacterial isolates from all the four vegetables of both of the sampling locations is illustrated in Fig. 3. Sequences were deposited in the GenBank and the accession numbers for each of the bacterial isolate along with details of their pathogen/non-pathogen nature (based on literature search) are presented in Table 2.

**Table 1:** Taxonomical classification of the bacterial isolates from salad vegetables

Isolate ID	Phylum	Class	Order	Family	Genus	Species
SBANHCA1, SBANHCa5, SBANHCa6, SBANHCa13, SBANHO7	Proteobacteria	Gammaproteobacteria	Xanthomonadales	Xanthomonadaceae	*Stenotrophomonas*	*maltophilia*
SBANHCA2, SBANHCa10, SBANHCa11, SBANHO2	Firmicutes	Coccus	Bacillales	Staphylococcaceae	*Staphylococcus*	*aureus*
SBANHCA3, SBANHCa4, SBANHT11	Proteobacteria	Gammaproteobacteria	Enterobacteriales	Enterobacteriaceae	*Salmonella*	*enterica*
SBANHCA7, SBANHCa9	Proteobacteria	Gammaproteobacteria	Enterobacteriales	Enterobacteriaceae	*Enterobacter*	*aerogenes*
SBANHCa8	Proteobacteria	Betaproteobacteria	Burkholderiales	Alcaligenaceae	*Bordetella*	*bronchiseptica*
SBANHCa12, SBANHCA15, SBANHCA17, SBANHCA15a, SBANHCa17a, SBANHO1, SBANHO4, SBANHO12, SBANSO81, SBANHT10, SBANHT12, SBANST3	Firmicutes	Bacilli	Bacillales	Bacillaceae	*Bacillus*	*pumilus*
SBANHCA14, SBANHCa1a	Proteobacteria	Gammaproteobacteria	Pseudomonadales	Pseudomonadaceae	*Pseudomonas*	*aeruginosa*
SBANHCA16	Firmicutes	Bacilli	Bacillales	Paenibacillaceae	*Paenibacillus*	*polymyxa*
SBANHCa16a	Firmicutes	Bacilli	Bacillales	Paenibacillaceae	*Paenibacillus*	*illinoisensis*
SBANHCa2a	Proteobacteria	Gammaproteobacteria	Enterobacteriales	Enterobacteriaceae	*Enterobacter*	*hormaechei*
SBANHCa7a, SBANHT9, SBANHT14, SBANHT15 SBANHCa14a	Firmicutes	Bacilli	Bacillales	Staphylococcaceae	*Staphylococcus*	*sciuri*
SBANSCa3	Firmicutes	Bacilli	Bacillales	Bacillaceae	*Bacillus*	*aerophilus*
SBANSCa4	Firmicutes	Bacilli	Bacillales	Bacillaceae	*Bacillus*	*cereus*
SBANSCa5	Actinobacteria	Actinobacteria	Actinomycetales	Microbacteriaceae	*Microbacterium*	*oleivorans*
SBANSCa6	Actinobacteria	Actinobacteria	Micrococcales	Micrococcaceae	*Arthrobacter*	*nicotianae*
SBANSCa7, SBANHT4, SBANST8, SBANST12	Firmicutes	Bacilli	Bacillales	Bacillaceae	*Bacillus*	*subtilis*
SBANSCa10, SBANSO82, SBANHT3	Firmicutes	Bacilli	Bacillales	Bacillaceae	*Bacillus*	*flexus*
SBANHCu23a, SBANHCu15, SBANHCu23, SBANHCu16, SBANHCu17, SBANHCu14a, SBANHCu15a, SBANHCu17a, SBANHT1, SBANHT16, SBANHT17	Proteobacteria	Gammaproteobacteria	Xanthomonadales	Xanthomonadaceae	*Stenotrophomonas*	*rhizophila*
SBANHCu24	Actinobacteria	Actinobacteria	Micrococcales	Micrococcaceae	*Arthrobacter*	*mysorens*
SBANHCu25a, SBANSCu19, SBANHCu19, SBANHCu22, SBANSCu22,	Proteobacteria	Gammaproteobacteria	Pseudomonadales	Pseudomonadaceae	*Pseudomonas*	*xanthomarina*
SBANHCu10, SBANHT21	Actinobacteria	Actinobacteria	Micrococcales	Promicromonosporaceae	*Cellulosimicrobium*	*cellulans*
SBANHCu12	Actinobacteria	Actinobacteria	Micrococcales	Microbacteriaceae	*Microbacterium*	*schleiferi*
SBANHCu14	Proteobacteria	Gammaproteobacteria	Xanthomonadales	Xanthomonadaceae	*Xanthomonas*	*axonopodis*
SBANHCu20, SBANHO3, SBANHO5, SBANHO8, SBANHO13, SBANHO14	Proteobacteria	Gammaproteobacteria	Pseudomonadales	Pseudomonadaceae	*Pseudomonas*	*stutzeri*
SBANHCu21, SBANSCu21	Proteobacteria	Betaproteobacteria	Burkholderiales	Alcaligenaceae	*Achromobacter*	*xylosoxidans*
SBANHCu24a	Actinobacteria	Actinobacteria	Micrococcales	Micrococcaceae	*Arthrobacter*	*protophormiae*
SBANHCu25	Proteobacteria	Gammaproteobacteria	Pseudomonadales	Pseudomonadaceae	*Pseudomonas*	*indoloxydans*
SBANHCu11	Proteobacteria	Gammaproteobacteria	Aeromonadales	Aeromonadaceae	*Aeromonas*	*hydrophila*
SBANSCu4	Firmicutes	Bacilli	Bacillales	Bacillaceae	*Geobacillus*	*stearothermophilus*
SBANSCu7	Firmicutes	Bacilli	Bacillales	Staphylococcaceae	*Staphylococcus*	*haemolyticus*
SBANSCu8	Firmicutes	Bacilli	Bacillales	Bacillaceae	*Bacillus*	*aerius*
SBANSCu9, SBANSCu11, SBANST7, SBANST11	Firmicutes	Bacilli	Bacillales	Bacillaceae	*Bacillus*	*megaterium*
SBANSCu10, SBANHCu12a, SBANSO80	Actinobacteria	Actinobacteria	Micrococcales	Microbacteriaceae	*Microbacterium*	*arborescens*
SBANSCu20	Proteobacteria	Gammaproteobacteria	Pseudomonadales	Pseudomonadaceae	*Pseudomonas*	*fluorescens*
SBANHO6	Proteobacteria	Gammaproteobacteria	Xanthomonadales	Xanthomonadaceae	*Xanthomonas*	*fuscans*
SBANHO9	Firmicutes	Bacilli	Bacillales	Listeriaceae	*Listeria*	*monocytogenes*
SBANHO11	Firmicutes	Bacilli	Bacillales	Bacillaceae	*Bacillus*	*anthracis*
SBANHO15	Firmicutes	Bacilli	Bacillales	Staphylococcaceae	*Staphylococcus*	*epidermidis*
SBANSO90, SBANHT7	Firmicutes	Bacilli	Bacillales	Staphylococcaceae	*Staphylococcus*	*gallinarum*
SBANSO54	Firmicutes	Bacilli	Lactobacillales	Enterococcaceae	*Enterococcus*	*faecium*
SBANSO83	Firmicutes	Bacilli	Bacillales	Bacillaceae	*Bacillus*	*aryabhattai*
SBANHT8	Firmicutes	Bacilli	Bacillales	Bacillaceae	*Geobacillus*	*stearothermophilus*
SBANHT13	Firmicutes	Bacilli	Bacillales	Bacillaceae	*Exiguobacterium*	*acetylicum*
SBANHT19	Actinobacteria	Actinobacteria	Micrococcales	Microbacteriaceae	*Microbacterium*	*testaceum*
SBANST6	Firmicutes	Bacilli	Bacillales	Bacillaceae	*Terribacillus*	*saccharophilus*
SBANST6a	Firmicutes	Bacilli	Bacillales	Bacillaceae	*Bacillus*	*tequilensis*

**Table 2:** Endophytic bacterial isolates from salad vegetables and their known characteristics

Bacterial isolate	Description^a^	Source vegetable	Isolate ID	Dilution	GenBank accession no.
*Stenotrophomonas maltophilia*	Opportunistic pathogen; associated with conjunctivitis, keratitis, scleritis, dacryrocystitis, cellulitis and endophthalmitis	Carrot, Carrot, Carrot, Carrot, Onion, Tomato	SBANHCA1, SBANHCa5, SBANHCa6, SBANHCa13, SBANHO7, SBANHT8	Crude, Crude, Crude, 10^-2^, Crude, Crude	KJ923784, KJ923788, KJ923789, KJ923796, KJ923776, KR186173
*Staphylococcus aureus*	Opportunistic pathogen; causes food poisoning, skin infection, pneumonia, septicemia, pyogenic infection, toxic shock syndrome and bacteremia	Carrot, Carrot, Carrot, Onion, Tomato,	SBANHCA2, SBANHCa10, SBANHCa11, SBANHO2, SBANHT3,	Crude, Crude, 10^-2^, Crude, Crude,	KJ923785, KJ923793, KJ923794, KJ923771, KR186184,
*Terribacillus saccharophilus*	Plant growth promoting rhizobacteria	Tomato	SBANST6	Crude	KR259242
*Bacillus tequilensis*	Plant growth promoting bacteria; nitrogen fixing	Tomato	SBANST6a	Crude	KR259246
*Salmonella enterica*	Highly pathogenic; causes food and water borne disease in human and animals; causes gastroenteritis, bacteraemia, enteric fever	Carrot, Carrot	SBANHCA3, SBANHCa4	Crude, Crude	KJ923786, KJ923787
*Enterobacter aerogenes*	Opportunistic pathogen; causes urinary tract infection, bacteraemia, endocarditis, septic arthritis, osteomyelitis and skin/soft tissue infection	Carrot, Carrot	SBANHCA7, SBANHCA9	Crude, Crude	KJ923790, KJ923792
*Bordetella bronchiseptica*	Opportunistic pathogen; causes respiratory tract infection in patients with cystic fibrosis and whooping cough	Carrot	SBANHCa8	Crude	KJ923791
*Bacillus pumilus*	Plant growth promoting bacteria; opportunistic pathogen; causes food poisoning and cutaneous infection in human.	Carrot, Carrot, Carrot, Carrot, Carrot, Onion, Onion, Onion, Onion, Tomato, Tomato, Tomato, Tomato, Tomato, Tomato	SBANHCa12, SBANHCA15, SBANHCa15a, SBANHCA17, SBANHCa17a, SBANHO1, SBANHO4, SBANHO12, SBANSO81, SBANHT1, SBANHT16, SBANHT17, SBANHT21, SBANST8, SBANST12	10^-2^, 10^-2^, 10^-2^, 10^-2^, 10^-2^, Crude, Crude, 10^-4^, Crude, Crude, 10^-2^, 10^-3^, 10^-4^, 10^-2^, 10^-2^	KJ923795, KJ923798, KR152298, KJ923800, KR152300, KJ923770, KJ923773, KJ923780, KR259236, KR186170, KR186180, KR186181, KR186183, KR259243, KR259245
*Pseudomonas aeruginosa*	Pathogenic; causes infection in immunocompromised individuals; causes endocarditis, osteomyelitis, pneumonia, urinary tract infection, gastrointestinal infections and meningitis	Carrot	SBANHCa1a, SBANHCA14	Crude, 10^-2^	KR152296, KJ923797
*Paenibacillus polymyxa*	Plant growth promoting bacteria; biocontrol agent and suppresses plant pathogens	Carrot	SBANHCA16	10^-2^	KJ923799
*Paenibacillus illinoisensis*	Plant growth promoting rhizobacteria; suppresses the activity of pathogens	Carrot	SBANHCa16a	10^-2^	KR152299
*Enterobacter hormaechei*	Causes nosocomial infection associated with blood stream infection	Carrot	SBANHCa2a	Crude	KR152301
*Staphylococcus sciuri*	Animal associated bacteria; causes endocarditis, peritonitis, septic shock, urinary tract infection, endophthalmitis, pelvic inflammatory disease and wound infection	Carrot, Carrot	SBANHCa7a, SBANHCa14a	Crude, 10^-2^	KR152302
*Bacillus aerophilus*	Bioremediation of imidacloprid, a synthetic insecticide	Carrot	SBANSCa3	Crude	KR186185
*Bacillus cereus*	Causes food poisoning; vomiting and diarrhoea	Carrot	SBANSCa4	Crude	KR186186
*Microbacterium oleivorans*	Plant beneficial bacteria; biocontrol agent to reduce mycotoxin in peanuts, grapes and cereals	Carrot	SBANSCa5	Crude	KR186187
*Arthrobacter nicotianae*	Environmentally beneficial bacteria; biodegradation of agro -chemicals	Carrot	SBANSCa6	Crude	KR186188
*Bacillus subtilis*	Plant beneficial bacteria; suppresses cucumber *Fusarium* wilt disease	Carrot	SBANSCa7	Crude	KR186189
*Bacillus flexus*	Plant endophytic bacteria; present in roots of wheat crop.	Carrot, Onion,	SBANSCa10, SBANSO82,	10^-2^, Crude,	KR186190, KR259237,
*Stenotrophomonas rhizophila*	Promotes plant growth; protects against biotic and abiotic stress; associated with human as a nosocomial pathogen	Cucumber, Cucumber, Cucumber, Cucumber, Cucumber, Cucumber, Cucumber, Cucumber	SBANHCu14a, SBANHCu15, SBANHCu15a, SBANHCu16, SBANHCu17, SBANHCu17a, SBANHCu23, SBANHCu23a	10^-2^, 10^-2^, 10^-3^ 10^-3^, 10^-4^, 10^-4^ 10^-4^, 10^-4^	KR259223, KR259224, KT020946, KR152303, KT020947, KR259225, KT020952, KR152304
*Arthrobacter mysorens*	Causes erythema with localised skin infection	Cucumber	SBANHCu24	10^-4^	KR152305
*Pseudomonas xanthomarina*	Arsenite oxidizing bacteria; helps in biodegradation of petroleum oil	Cucumber, Cucumber, Cucumber, Cucumber, Cucumber	SBANHCu19, SBANHCu22, SBANHCu25a, SBANSCu19, SBANSCu22	10^-3^, 10^-4^, 10^-4^, 10^-2,^ 10^-4^	KT020948, KT020951, KR152306, KR259226, KR259229
*Cellulosimicrobium cellulans*	Opportunistic pathogen; causes catheter related bacteraemia with short bowel syndrome in children, peritonitis, endocarditis and joint, ocular and soft-tissue infections	Cucumber	SBANHCu10	Crude	KT020943
*Microbacterium schleiferi*	Environmentally beneficial bacteria; helps in bioremediation of 1, 3, 5 – TMB (trimethylebenzene)	Cucumber	SBANHCu12	10^-2^	KT020944
*Xanthomonas axonopodis*	Plant pathogenic bacteria; causes bacterial pustule disease in soybean	Cucumber	SBANHCu14	10^-2^	KT020945
*Pseudomonas stutzeri*	Opportunistic pathogen; causes community acquired pneumonia, meningitis, neonatal septicaemia and knee arthritis in children	Cucumber, Onion, Onion, Onion, Onion, Onion, Tomato	SBANHCu20, SBANHO3, SBANHO5, SBANHO8, SBANHO13, SBANHO14, SBANHT4	10^-3^, Crude, Crude, 10^-2^, 10^-3^, 10^-3^, Crude	KT020949, KJ923772, KJ923774, KJ923777, KJ923781, KJ923782, KR186171
*Achromobacter xylosoxidans*	Opportunistic pathogen; causes malignancies, cardiac disease, meningitis, urinary tract infections, abscesses, osteomyelitis, corneal ulcers, prosthetic valve endocarditis, peritonitis and pneumonia	Cucumber, Cucumber	SBANHCu21, SBANSCu21	10^-3^, 10^-4^	KT020950, KR259228
*Arthrobacter protophormiae*	Opportunistic pathogen; present in human skin as commensals; widely distributed in pesticide contaminated agricultural fields	Cucumber	SBANHCu24a	10^-4^	KT020953
*Pseudomonas indoloxydans*	Useful in biological synthesis of indigo, an important dye-stuff used in textile industries	Cucumber	SBANHCu25	10^-4^	KT020954
*Aeromonas hydrophila*	Opportunistic pathogen; causes enteritis, wound infection, septicaemia, pneumonia and conjunctivitis	Cucumber	SBANHCu11	Crude	KU564062
*Geobacillus stearothermophilus*	Persists as contaminant in canned food; used as biological indicator for deactivation of pathogens	Cucumber	SBANSCu4	Crude	KR259230
*Staphylococcus haemolyticus*	Highly pathogenic; most frequently isolated from blood culture	Cucumber	SBANSCu7	Crude	KR259231
*Bacillus aerius*	Halophilic endophytic bacteria; plant growth promotion, phosphate solubilisation, bioremediation of salt affected soil	Cucumber	SBANSCu8	Crude	KR259232
*Bacillus megaterium*	Plant beneficial bacteria; helps in nitrogen fixation and promotes plant growth	Cucumber, Cucumber	SBANSCu9, SBANSCu11	Crude, 10^-2^	KR259220, KR259233,
*Microbacterium arborescens*	Rhizosphere bacteria of sand dune plant	Cucumber, Cucumber, Onion, Tomato	SBANSCu10, SBANHCu12a, SBANSO80, SBANHT19	Crude, 10^-2^, Crude, 10^-4^	KR259221, KR259222, KR259235, KR186182
*Pseudomonas fluorescens*	Plant beneficial rhizobacteria; causes red skin disease with skin hemorrhage and ulceration	Cucumber	SBANSCu20	10^-3^	KR259227
*Xanthomonas fuscans*	Plant pathogenic bacteria; infects rice, banana, citrus, bean, tomato, pepper, sugarcane and wheat	Onion, Tomato	SBANHO6, SBANHT7	Crude, Crude	KJ923775, KR186172
*Listeria monocytogenes*	Food borne pathogen; causes bacterial meningitis, multiple cerebral ring enhancing lesions	Onion, Tomato, Tomato, Tomato	SBANHO9, SBANHT9 SBANHT14, SBANHT15	10^-2^, Crude, 10^-2^, 10^-2^	KJ923778, KR186174, KR186178, KR186179
*Bacillus anthracis*	Obligate pathogen; causes anthrax: inhalation, gastrointestinal and cutaneous	Onion, Tomato, Tomato	SBANHO11, SBANHT10, SBANHT12	10^-2^, Crude, 10^-2^	KJ923779, KR186175, KR186176
*Staphylococcus epidermidis*	Opportunistic pathogen; causes cardiovascular, CNS shunts, joints, blood stream infection and nosocomial infection	Onion,	SBANHO15,	10^-3^,	KJ923783,
*Exiguobacterium acetylicum*	Plant beneficial bacteria; in human it causes catheter related bacteraemia	Tomato	SBANHT13	10^-2^	KR186177
*Staphylococcus gallinarum*	Rare human pathogen; causes abdominal pain, nausea and weakness	Onion, Tomato	SBANSO90, SBANHT11	10^-3^, 10^-2^	KR259239, KU564061
*Enterococcus faecium*	Opportunistic pathogen; causes urinary tract, wound and soft tissue infections, bacteraemia, endocarditis	Onion, Tomato	SBANSO54, SBANST3	Crude, Crude	KR259234, KR259241
*Bacillus aryabhattai*	Plant growth promoting bacteria	Onion, Tomato, Tomato	SBANSO83, SBANST7, SBANST11	10^-2^, Crude, 10^-2^	KR259238, KR259240, KR259244

^a^ References of description are given in discussion section

The diversity indices of the endophytic bacterial isolates from the four salad vegetables were calculated using the PAST program (Table 3). The diversity indices were calculated to obtain the diversity of bacterial isolates in the salad vegetables which revealed a lower diversity in tomato, whereas carrot had the highest bacterial diversity compared to other salad vegetables. Moreover, the Dominance (D) and Berger—Parker indices clearly showed that the single taxa of the salad vegetables (tomato) were abundant in the community and the number of isolates are shown in Table 3.

**Table 3:** Diversity indices of endophytic bacterial population of the salad vegetables

Diversity indices/parameters	Formula	Carrot	Cucumber	Onion	Tomato
Taxa (S)	-	5	4	4	3
Individuals (n)	-	31	30	20	21
Dominance (D)	D = Sum (n_i_/n)^2^	0.3403	0.3933	0.43	0.61
Simpson (1-D)	1 – D = 1 - Sum (n_i_/n)^2^	0.6597	0.6067	0.57	0.39
Shannon (H)	H = Sum ((n_i_/n) In (n_i_/n)^)^	1.245	1.123	0.9958	0.7091
Evenness (E)	E = e^H^/S	0.6949	0.7684	0.6767	0.6774
Brillouin (HB)	HB = In (n) – Sum In (n_i_)	1.067	0.9698	0.8154	0.5821
Menhinick (db)	db = S/ $\sqrt{n}$	0.898	0.7303	0.8944	0.6547
Margalef (Ma)	Ma = (S-1)/In (n)	1.165	0.882	1.001	0.6569
Equitability (J)	J = H/Hmax	0.7738	0.81	0.7183	0.6455
Fisher alpha (FA)	S = α*In (1 + n/α)	1.687	1.24	1.504	0.9578
Berger-Parker (d)	d = n/nT	0.4194	0.5667	0.55	0.7619
Chao-1	Chao1 = S + F_1_(F_1_ - 1)/(2 (F_2_ + 1)),	5	4	5	3

*n* = number of individuals; *n*_i_ = number of individuals of taxon i; S = number of taxa; nT = number of individuals in the dominant taxon

Hmax = log S

**Fig. 4:**
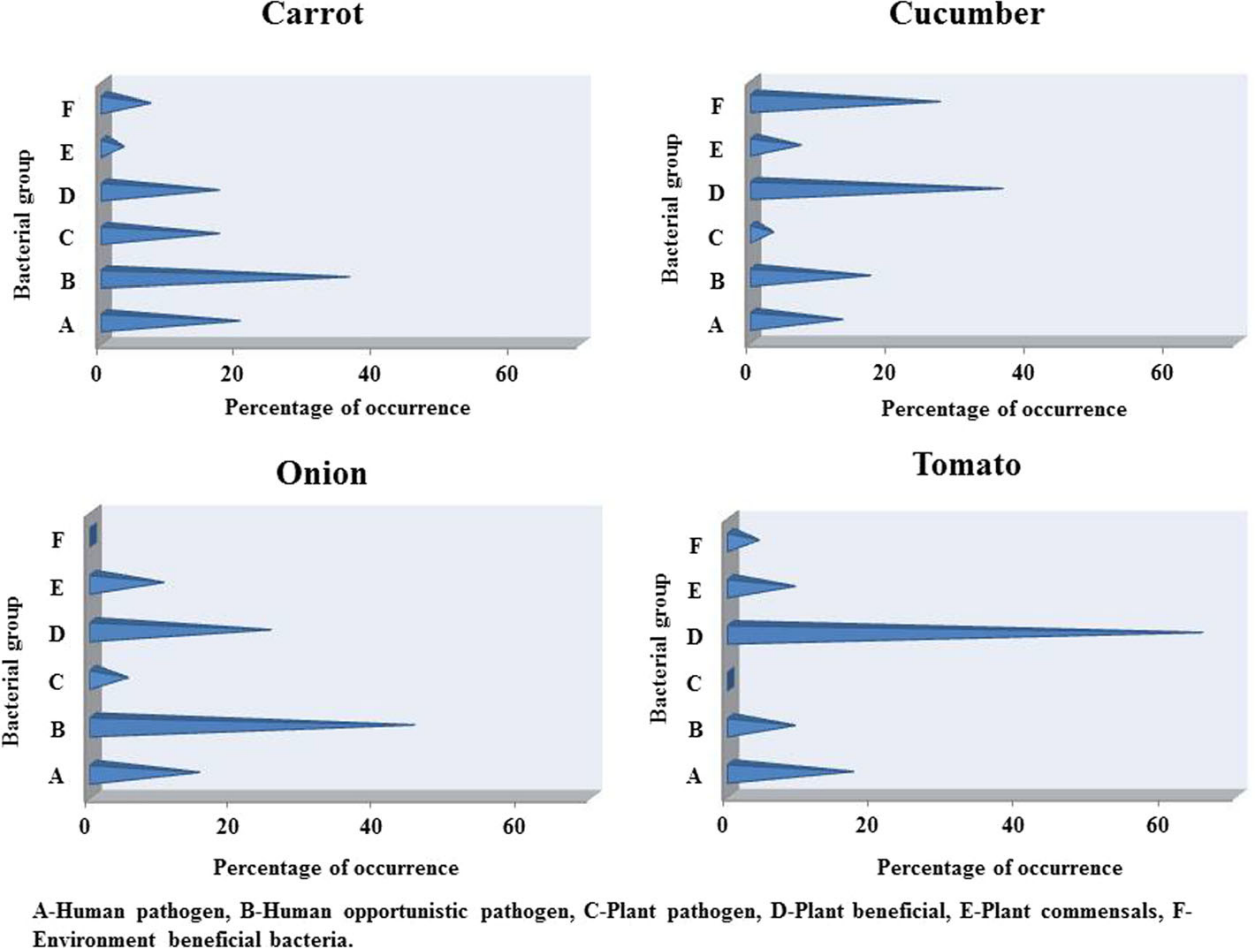
Classification of endophytic bacteria in salad vegetables based on pathogen/non-pathogen nature

The isolates were further classified as human pathogens, human opportunistic pathogens, plant pathogens, plant beneficial, plant commensal and environmentally beneficial bacteria and represented as percentage occurrence in each vegetable type (Fig. 4). For this categorization, repeated occurrence of the same species was also counted and represented as percentage to the total population in a given vegetable. It was observed that human opportunistic pathogen was predominant in carrot and onion, whereas plant beneficial bacteria dominated in cucumber and tomato.

## Discussion

In spite of potential beneficial aspects of salad vegetables, concerns over their safety and quality have risen since there are number of existing factors responsible for contamination. Fresh salad vegetables have little or no processing steps that can reduce the pathogen levels.

In general, plant harbours diverse bacterial populations both as epiphytes and endophytes, and they are classified based on their characteristics as plant associated bacteria, plant pathogenic, plant growth promoting bacteria and human pathogenic. The presence of bacterial populations as endophytes cannot be removed by washing and thus enters human during consumption of fresh produce as raw salads [9]. Vegetables are considered as the major reservoirs of opportunistic and emerging pathogens due to its diverse microbiome and they are also strongly influenced by biogeographic aspects of farming and food processing practices [10].

In United States, the importance and of fresh produce related infection was realized after the onset of shiga toxin producing *E. coli* outbreak related to fresh vegetables [11]. The ready-to-eat foods of non-animal origin such as, fruits, vegetables, salads, seeds, nuts, cereals, herbs, spices, fruits and algae had been reported to cause several foodborne outbreaks between 2007 to 2011, in which the major outbreak was with the combination of *Salmonella* spp. with leafy greens, bulb and stem of vegetables tomatoes and melons, and *E. coli* with fresh pods, legumes and grains [12]. Consumption of fresh produce raised the occurrence of gastroenteritis, caused by *E. coli* 0157:H7 and non-typhoidal *Salmonella* which were traditionally considered as non-hosts for human enteric pathogens. The occurrence of produce-associated outbreaks highlighted our deficiencies in understanding the ecology of enteric pathogens outside human and animal host [13].

The endophytic bacterial isolates observed in our study in tomato, onion, cucumber and carrot belonged to actinobacteria, firmicutes, betaproteobacteria, gammaproteobacteria, bacilli and cocci. The bacteria were categorized based on the known characteristics such as pathogens, non-pathogens and beneficial. Previous reports on the occurrence of human enteric pathogens in salad vegetables were based mostly on enrichment cultures. The bacterial isolates we obtained in the present study were either from crude extract or dilutions up to 10^-4^. Without enrichment, the average percentage of human pathogens obtained in salad vegetables was 16.25% and of human opportunistic pathogens were 26.5% of the total endophytic isolates. This represents high level of contamination with pathogenic bacteria in the salad vegetables studied. In India, particularly in Tamil Nadu, domestic sewage, industrial and municipal waste water is used for irrigating vegetable crops. This could be one of the reasons for the human pathogenic bacterial load observed in salad vegetables, in addition to possible other reasons including contamination through human and farm animal waste in agricultural lands, post-harvest handling, transport, storage and poor hygiene conditions prevailing in market places.

Viswanathan and Kaur [14] has reported existence of *P. aeruginosa*, *E. coli*, *S. aureus*, *Enterobacter* sp., *Salmonella* sp. in carrot. *E. coli* and *S. enterica* are known to adapt and persist in plant environment and increase the chance of transmitting to humans *via* consumption of plants or plant-derived products [15]. The ready-to-eat salads and ready-to-eat sprouts are known to be major vehicle for non-tuberculosis *Mycobacteria* transmission in humans [16]. Endophytic microorganisms associated with carrot, cucumber, cabbage and onions were analysed by Tayo et al. [1] and *E. coli* was found to be more predominant, followed by *Enterobacter* sp., *S. aureus*, *Erwinia* sp., *Pseudomonas* sp. and *Salmonella* sp.

Among the bacteria that were identified in our study across four vegetables, major genera were *Bacillus* followed by *Staphylococcus*, *Pseudomonas* and *Microbacterium*. Occurrence of *Bacillus aerius* was unique to cucumber, similarly *B. tequilensis* to tomato, *B. anthracis* and *B. aryabhattai* to onion. *B. pumilus* and *B. flexus* were found to occur in carrot, onion and tomato. We have previously reported the endophytic colonization of *B. pumilus* in apples, *B. flexus* and *B. subtilis* in oranges [17]. *B. aryabhattai* [18], *Terribacillus saccharophilus* [19] and *B. tequilensis* [20] are bacterial strains isolated from the upper atmospheric air of field soils in Japan and 2000-year old Mexican shaft-tomb. *B. cereus* is an opportunistic pathogen causing food poison [21]. *B. anthracis* is an obligate pathogen, which survive in several environmental conditions as saprobionts and known to be involved in horizontal gene transfer in plant rhizosphere [22]. *Geobacillus stearothermophilus* produces lactic acid by fermenting potato starch and potato residues [23]. *Paenibacillus illinoisensis* has been reported to suppress the activity of antioxidative enzymes in pepper roots caused by *Phytophthora capsici* infection [24].

In the present study, *Staphylococcus sciuri* was found to occur only in carrot, *S. epidermidis* in onion and *S. haemolyticus* in cucumber. *S. aureus* was obtained from carrot as well as onion and *S. gallinarium* from onion as well as tomato. *S. aureus* is considered as a commensal and a pathogen in human [25]. *S. sciuri*, animal associated bacteria, is known to be present widely on the skin and mucous surface of pet and farm animals and also in soil, sand, water and marsh grass. The presence of *S. sciuri*, its adaptation and continuous existence in hospital environment has also been reported [26]. *S. epidermidis* has been reported as an important human opportunistic pathogen causing infection in cardiovascular, CNS shunts, joints, blood stream etc. [27]. *S. haemolyticus* is an important hospital acquired pathogen, frequently isolated from human blood cultures [28]. *S. gallinarum*, a rare human pathogen has been isolated from chicken and saliva of healthy humans [29].

Four among the five species of *Pseudomonas* observed in our study (*P. aeruginosa, P. xanthomarina, P. indoloxydans* and *P. fluorescens*) were from cucumber only. *P. stutzeri* was found in cucumber and onion. *Pseudomonas fluorescens* is known to display plant growth promoting rhizobacterial (PGPR) activity [30]. It has been isolated from rhizosphere soils of many crop plants and has received the most research attention among the known PGPR genera. In human, it is a less virulent bacterium causing nosocomial infections in immune-compromised patients [31]. Hu et al. [32] has reported it as a common aquaculture pathogen. *P. stutzeri* causes knee arthritis in children [33]. *P. xanthomarine* has been reported as a novel bacteria isolated from marine ascidian [34] and from arsenic contaminated soil [35]. *P. stutzeri* and *P. xanthomarine* has the highest biodegrading ability to degrade crude petroleum oil [36]. *P. indoloxydans* is a lindane (chlorinated pesticide) degrading bacteria isolated from soils of lindane manufacturing industrial site [37].

We also observed *Microbacterium oleivorans* in carrot, *M. schleiferi* in cucumber, *M. testaceum* in tomato and *M. arborescens* in cucumber and onion. *M. testaceum* [38] is an environment friendly bacterium which helps in improving the plant growth and yield of crops by suppressing the growth of plant pathogens. *M. oleovorans* has been reported as a biocontrol agent against *Fusarium verticillioides* and *Aspergillus flavus* [39].

*Cellulosimicrobium cellulans* which was observed in cucumber and tomato tissues is a rare human pathogen, causing septic arthritis mainly in immune-compromised patients [40]. In cucumber plants, it supresses the self-toxic effect by degrading cinnamic acid, benzoic acid, paraaminobenzoic acid and phenol, helps in nutrient absorption rate, adjusts microbial groups in non-rhizospheric soil, thus increasing the number of beneficial bacteria and decreasing fungal growth [41]. Thus, in spite of being a human pathogen, *C. cellulans* can also act as biocontrol agent for plant pathogens. *Stenotrophomonas maltophila* has been reported from cucumber rhizosphere involved in supressing *Phytophthora* crown rot [42]. We observed *S. maltophila* in carrot and *S. rhizophila* in cucumber and tomato indicating the species specificity of the bacteria according to the vegetable specific endosphere.

*Salmonella enterica* is an important foodborne human pathogen, which has the ability to colonize number of plant species such as, Arabidopsis, alfalfa, tomato and various leafy green vegetables [43]. *Salmonella* triggers mutation in pathogen associated molecular patterns (PAMP) motifs to escape plant defence and it also expresses effectors in the plant tissues to manipulate the plant immune system by triggering PTI (PAMP- triggered immunity) to evolve strategies to avoid or subvert plant immunity [43]. An increase in the growth of *S. enteritidis* has been reported in melon, watermelon and papaya at different temperatures (10, 20 and 30 °C) [44]. The proliferation of *Salmonella* is high on red ripped tomato tissues compared to green tomato tissues [45]. It attaches to stem and flowers of tomato plant and remains viable during development of fruit and serves as a route cause for the contamination of tomato fruits [46].

*Listeria monocytogenes*, *Aeromonas hydrophila* and *Clostridium botulinum* have been considered as the most notable pathogens which maintain their infection potential under mild preservation conditions. *A. hydrophila* has the potential to grow at 0 °C and the temperature of 4–5 °C supports the growth in foods. *L. monocytogenes* survives in ready-to-eat fresh salads and causes public health risk [47]. *Exiguobacterium acetylicum* has been reported as an aetiological agent of bacteraemia in humans [48]. Whereas in wheat plants, it improves the growth of seedlings and inhibits the growth of plant pathogens and it is also a cold tolerant bacteria [49]. *Bordetella bronchiseptica* infects mainly immune-suppressed populations of humans and causes respiratory tract related disease in dogs, cats and rabbits [50]. *Arthrobacter protophormiae*, when pre-exposed to lower concentration of ONB (O- nitrobenzoate) and PHB (p- hydroxybenzoate) will change its cellular fatty acid profile and adapt itself to survive in high concentration of ONB and PHB [51]. *A. mysorens* causes erythema characterized by localized skin infection [52]. *A. xylosoxidans* was reported to be associated with number of outbreaks related to various infections such as malignancies, cardiac disease, meningitis, endocarditis, and pneumonia with number of death cases [53]. *Enterobacter hormaechei* a unique species was first identified in 1989 followed by several outbreaks on sepsis in neonatal intensive care units in Brazil and USA [54]. *E. aerogenes* and *E. cloacae* has been reported in hospital acquired infections in Europe and France, and they also have strong antibiotic resistance, which helps in colonizing multiple environments and hosts [55]. *E. asburiae*, epiphytic bacteria, exists as parasitic or commensal with in plant host and produce virulence factors based on their quorum sensing activity and has been isolated from soil, water, food products and lettuce leaves [56]. The type III secretion system (T3SS) encoded by *hrp* gene in plant associated bacteria *Xanthomonas fuscans* sub sp. *fuscans* is responsible for colonizing bean plants and causing bacterial blight disease [57]. Plant pathogenic bacterium *Xanthomonas axonopodis* cause bacterial pustule disease in soybean [58].

Surette et al. [59] enumerated the endophytic bacteria in carrot and found 83% of the strains were plant growth promoting bacteria. In our study, carrot contained more of human opportunistic pathogens followed by human pathogens, plant beneficial and plant pathogenic bacteria. In carrot, population of human opportunistic pathogens were high and approximately double the number of plant pathogens and plant beneficial bacteria. The scenario was the other way around in cucumber, where plant beneficial bacterial population was double and more than double the population of human opportunistic and human pathogens, respectively. In onion, human opportunistic pathogen population was high compared to plant beneficial. Tomato had a comparatively very high population of beneficial bacteria in which the human pathogens and plant pathogens were found to be drastically reduced. Comparing the functional diversity in all four vegetables, existence of high population of plant beneficial bacteria has suppressed the growth of human pathogens, human opportunistic pathogens and plant pathogens. Whether the difference in bacterial population between pathogens and non-pathogens is due to the suppression effect of plant beneficial bacteria on human opportunistic pathogens or suppression effect of human opportunistic pathogens on plant beneficial bacteria needs further in-depth analysis of their interaction in this ecological niche.

It is also interesting to note that population of human pathogens remain more or less similar (approx. 20% to the total) in carrot and tomato, irrespective of reasonably high occurrence of human opportunistic pathogens (carrot) and plant beneficial bacteria (tomato). However, the occurrence of human pathogens was below 20% when the combined population of human opportunistic pathogens and plant beneficial bacteria (onion) or plant beneficial and environmentally beneficial bacteria (cucumber) was above 50%. Percentage of human pathogens was less than 20%, only when population of plant beneficial bacteria was above 20%. Population of human opportunistic pathogens was 45, 36, 17 and 8% respectively in onion, carrot, cucumber and tomato compared to the population percentage of 25, 17, 36 and 63 of plant beneficial bacteria in these vegetables. There is a clear evidence for the suppressive effect of plant beneficial bacteria on human opportunistic pathogens in cucumber and tomato. When the population of plant beneficial bacteria was above 17% (onion – 25; cucumber – 36; tomato – 63), the plant pathogens were accordingly suppressed (onion – 5; cucumber – 3; tomato – 0). Although pathogen population was less (5%) due to higher number of human opportunistic pathogens (45%), as observed in onion, similar trend was not observed in cucumber and tomato where the suppression of plant pathogens (3 and 0% respectively) was parallel to human opportunistic pathogens (17 and 8% respectively). This indicates the suppressed population of these two groups of bacteria are independent of each other and dependent on plant beneficial bacteria. It can be concluded that plant beneficial bacteria suppresses both plant and human opportunistic pathogens. Occurrence of commensal bacteria lacks correlation with population of other categories of bacteria. However, commensal bacteria were found to be suppressed when all other bacteria contribute reasonably to the total population as observed in carrot. Higher occurrence of human opportunistic bacteria (45% in onion) was found to have a significant effect on environmentally beneficial bacteria. Previous reports indicate the difference in composition of plant microbiome based on the soil contamination with pollutants as well as organic cultivation and pesticide usage [60, 61]. Hence, the observed composition of the vegetable bacteriome would be due to the prevailing soil conditions such as pollutant and pesticide level. Further studies on the bacteriome of these vegetables from different agro-climatic zones, soils and cultivation conditions would be useful in understanding the basal level bacterial species specific to these vegetables. In addition, such studies will also help to identify soil, climatic zone and pollutant specific species which have gained endophytic status.

## Conclusions

The complex microbial community analysis and their interactions with plant have greater potential to elucidate interactions between host plants and bacteria as well as bacteria—bacteria interactions. Elucidating the cross-talk between different bacterial communities would not only help in understanding the interaction but also to evolve new biocontrol agents for plant and human pathogens. Based on the observations on relative population density of different categories of the bacteriome in salad vegetables, we propose that there is a greater potential to study the native endophytic plant beneficial bacteria and to develop them as biocontrol agents against not only the plant pathogens, but also the human pathogens that are harboured by plants.

## Additional file

Additional file 1: Table S1. (a). Morphological and biochemical characterization of bacterial isolates from carrot. (b). Morphological and biochemical characterization of bacterial isolates from cucumber. (c). Morphological and biochemical characterization of bacterial isolates from onion. (d). Morphological and biochemical characterization of bacterial isolates from tomato. (DOC 233 kb)

## Abbreviations

AIA: *Aeromonas* Isolation Agar
BLAST: Basic Local Alignment Search Tool
C: Citrate
CA: Certemaid Agar
CDC: Center for Disease Control and Prevention
CFU: Colony Forming Units
CNS: Central Nervous System
CSPI: Center for Science in the Public Interest
EMBA: Eosin Methylene Blue Agar
FDA: US Food and Drug Administration
I: Indole
M: Motility
MR: Methyl Red
MSA: Mannitol Salt Agar
N: Nitrate
O: Oxidase
ONB: O- nitrobenzoate
PAMP: Pathogen Associated Molecular Patterns
PHB: P- hydroxybenzoate
PTI: PAMP- triggered immunity
SSA: *Salmonella*—*Shigella* Agar
T3SS: Type III Secretion System
TAL: Transcription Activator Like effector
VP: Voges—Proskauer
WHO: World Health Organization

## References

[1] Tayo BC, Odu NN, Esen CU, Okonko IO (2012). Microorganisms associated with spoilage of stored vegetables in Uyo Metropolis, Akwa Ibom state, Nigeria. Nat Sci.

[2] Vaz BM, Fink RC, Gonzalez FD, Sadowsky MJ (2014). Minireview enteric pathogen-plant interactions: molecular connections leading to colonization and growth and implications for food safety. Microbes Environ.

[3] Leff JW, Fierer N (2013). Bacterial communities associated with the surfaces of fresh fruits and vegetables. PLoS One.

[4] Rovira J, Cencic A, Santos E, Jakobsen M, Luning PA, Devlieghere F, Verhe R (2006). Biological hazards. Safet in the agri–food chain.

[5] Wiedemann A, Payant IV, Chausse AM, Schikora A, Velge P (2015). Interactions of *Salmonella* with animals and plants. Front Microbiol.

[6] Saitou N, Nei M (1987). The neighbor-joining method: A new method for reconstructing phylogenetic trees. Mol Biol Evol.

[7] Tamura K, Stecher G, Peterson D, Filipski A, Kumar S (2013). MEGA6: molecular evolutionary genetics analysis version 6.0.. Mol Biol Evol.

[8] Hammer O, Harper DAT, Rayn PD (2001). PAST: Paleontological statistics softer pakage for education and data analysis. Palaeontol Electronic.

[9] Jackson CR, Randolph KC, Osborn LO, Tyler HL (2013). Culture dependent and independent analysis of bacterial communities associated with commercial salad leaf vegetables. BMC Microbiol.

[10] Berg G, Erlacher A, Smalla K, Krause R (2014). Vegetables microbiomes is there a connection among opportunistic infections, human health and our gut feeling?. Microb Biotechnol.

[11] Rivas M, Chines I, Miliwebsky E, Masana M (2014). Risk factors for Shiga toxin –producing *Escherichia coli* associated human diseases. Microbiol Spectrum.

[12] Felicio MIDS, Hald T, Liebana E, Allende A, Hugas M, Johannersen GK, Niskanen T, Uyttendaele M, Mchauchlin J, The CN (2015). Risk ranking pathogens in ready-to-eat unprocessed foods of non – animal origin (FONAO) in the EV: Initial evaluation using outbreak data (2007-2011). Int J Food Microbiol.

[13] Teplitski M, Barak JD, Schneider KR (2009). Human enteric pathogens in produce :un-answered ecological questions with direct implications for food safety. Curr Opin Biotechnol.

[14] Viswanathan P, Kaur R (2001). Prevalence and growth of pathogens on salad vegetables, fruits and sprouts. Int J Hyg Environ Health.

[15] Overbeek LSV, Doom JV, Wichers JH, Amerongen AV, Roermund HJWV, Willemsen PTJ (2014). The arable ecosystem as bottle ground for emergence of new human pathogens. Front Microbiol.

[16] Cortes JFC, Monter NL, Cueto ALC, Rangel LPS, Reptto ACH, Hernandez DL, Gutierrez SR, Rendon EF, Merchand JAG. Microbiological Quality of ready-to-eat vegetables collected in Mexico city: Occurrence of aerobic-mesophilic bacteria, fecal coliforms and potentially pathogenic nontuberculous *Mycobacteria*. BioMed Res Int. 2015;2015:789508. 10.1155/2015/789508 PMC4396000 25918721

[17] Phukon M, Sahu P, Srinath R, Nithya A, Babu S (2013). Unusual occurance of *Staphylococcus warneri* as endophyte in fresh fruits along with usual *Bacillus* spp. J Food Safety.

[18] Shivaji S, Chaturvedi P, Begum Z, Pindi PK, Manorama R, Padmanaban A, Shouche YS, Pawar S, Vaishampayan P, Dutt CBS, Datta GN, Manchanda RK, Rao UR, Bhargava PM, Narlikar JV (2009). *Janibacter hoylei* sp. nov., *Bacillus isronensis* sp. nov. and *Bacillus aryabhattai* sp. nov., isolated from cryotubes used for collecting air from the upper atmosphere. Int J Syst Evol Microbiol.

[19] An SY, Asahara M, Goto K, Kasai H, Yokota A (2007). *Terribacillus saccharophilus* gen. nov., sp. Nov. and *Terribacillus halophilus* sp. nov., spore-forming bacteria isolated from field soil in Japan. Int J Syst Evol Microbiol.

[20] Gatson JW, Benz BF, Chandrasekaran C, Satomi M, Venkateswaran K, Hart ME (2006). *Bacillus tequilensis* sp. nov., isolated from a 2000-year-old Mexican shaft-tomb, is closely related to *Bacillus subtilis*. Int J Syst Evol Microbiol.

[21] Raymond B, Wyres KL, Sheppard SK, Ellis RJ, Bonsall MB (2010). Environmental factors determining the epidemiology and population genetic structure of *Bacillus cereus* group in the field. PLoS Pathog.

[22] Ganz HH, Turner WC, Brodie EL, Kusters M, Shi Y, Sibanda H, Torok T, Getz WM (2014). Interactions between *Bacillus anthracis* and plants may promote anthrax transmission. PLoS Negl Trop Dis.

[23] Smerilli M, Neureiter M, Wurz S, Haas C, Fruhauf S, Fuchs W (2015). Direct fermentation of potato starch and potato residues to lactic acid by *Geobacillus stearothermophilus* under non-sterile conditions. J Chem Technol Biotechnol.

[24] Jung WJ, Jin YL, Park RD, Kim KY, Lim KT, Kim TM (2006). Treatment of *Paenibacillus illinoisensis* suppresses the activities of antioxidative enzyme in pepper roots caused by *Phytophthora capsici* infection. World J Microbiol Biotechnol.

[25] Chaves-Moreno D, Wos-Oxley ML, Jauregui R, Medina E, Oxley APA, Pieper DH. Exploring the transcriptome of *Staphylococcus aureus* in its natural niche. Sci Rep. 2016; 6. doi: 10.1038/srep33174. 10.1038/srep33174 PMC5027550 27641137

[26] Dakic I, Morrison D, Vukovic D, Savic B, Shittu A, Jezek P, Hauschild, Stepanovic S (2005). Isolation and molecular characterization of *Staphylococcus sciuri* in the hospital environment. J Clin Microbiol.

[27] Buttner H, Mack D, Rohde H (2015). Structural basis of *Staphylococcus epidermidis* biofilm formation: mechanisms and molecular interactions. Front Cell Infect Microbiol.

[28] Barros EM, Ceotto H, Bastos MCF, Santos KRN, Giambiagi M (2011). *Staphylococcus haemolyticus* as an important hospital pathogen and carrier of methicillin resistance genes. J Clin Microbiol.

[29] Tibra NK, Jalali S, Reddy AK, Narayanan R, Agarwal R (2010). Traumatic endophthalmitis caused by *Staphylococcus gallinarum*. J Med Microbiol.

[30] Santoro MV, Bogino PC, Nocelli N, Cappellari LR, Giordano WF, Banchio E. Analysis of plant growth-promoting effects of fluorescent Pseudomonas strains isolated from *Mentha piperita* rhizosphere and effects of their volatile organic compounds on essential oil composition. Front Microbiol. 2016. http://dx.doi.org/10.3389/fmicb.2016.01085. 10.3389/fmicb.2016.01085 PMC4949228 27486441

[31] Wong V, Levi K, Baddal B, Turton J, Boswell TC (2011). Spread of *Pseudomonas fluorescens* due to contaminated drinking water in a bone marrow transplant unit. J Clin Microbiol.

[32] Hu YH, Dang W, Sun L (2012). The ton B- dependent outer membrane receptor of *Pseudomonas fluorescens*: virulence and vaccine potential. Arch Microbiol.

[33] Miron D, Keness Y, Bor N, Spiegel R, Horowitz Y (2007). *Pseudomonads stutzeri* knee arthritis in a child: case report and review. J Pediatr Orthop B.

[34] Romanenko LA, Uchino M, Falsen E, Lysenko AM, Zhukova NV, Mikhailov VV (2005). *Pseudomonas xanthomarine* sp. a novel bacterium isolated from marine ascidian. J Gen Appl Microbiol.

[35] Koechler S, Ploetze FA, Armanet CB, Chollet FG, Salmeron AH, Jost B, Lievremont D, Philipps M, Plewniak F, Bertin PN, Lett MC (2015). Constituvative arsenite oxidase expression detected in arsenic-hypertolerant *Pseudomonas xanthomarine* S11. Res Microbiol.

[36] Sheshtawy HSE, Khalil NM, Ahmed W, Abdallah RI (2014). Monitoring of oil pollution at Gemsa Bay and bioremediation capacity of bacterial isolates with bio-surfactants and nanoparticles. Marine Poll Bull.

[37] Manickam N, Ghosh A, Jain RK, Mayilraj S (2008). Description of a novel indole-oxidizing bacteria *Pseudomonas indoloxydans* sp. nov., isolated from pesticide-contaminated site. Syst Appl Microbiol.

[38] Morohoshi T, Wang WZ, Someya N, Ikeda T (2011). Genome sequence of Microbacterium testaceu StLB037, an N-Acylhomoserine Lactone-Degrading bacterium isolated from potato leaves. J Bacteriol.

[39] Chulze SN, Palazzini JM, Torres AM, Barros G, Ponsone ML, Geisen R, Schmidt HM, Kohl J (2015). Biological control as a strategy to reduce the impact of mycotoxins in peanut, grapes and cereals in Argentina. Food Addit Contam Part A.

[40] Checa CM, Chaparro LC, Ruiz JP, Monje AP, Alexander JLR, Salvatierra J, Raya E (2011). Septic arthritis due to *Cellulosimicrobium cellulans*. J Clin Microbiol.

[41] Yu GH, Xiey H, Chen YH, Chen YF, Cheng P (2006). Mitigating the repress of cinnamic acid to cucumber growth by microbial strain. Wei Sheng Wu Xue Bao.

[42] Islam S, Akanda AK, Prova A, Islam MT, Hossaim MM (2016). Isolation and identification of plant growth promoting rhizobacteria from cucumber rhizosphere and their effect on plant growth promotion and disease suppression. Front Microbiol.

[43] Garcia AV, Charrier A, Schilcora A, Bigeard J, Pateyron S, Moreau MLT, Evrard A, Mithofer A, Magniette MLM, Payant IV, Hirt H (2014). *Salmonella enteric* flagellin is recognized *via* FLS2 and activities PAMP-triggered immunity in *Arabidopsis thaliana*. Mol Plant.

[44] Penteado AL, Leitao MFF (2004). Growth of *Salmonella* Enteritidis in melon, water melon and papaya pulp stored at different time and temperature. Food Control.

[45] Marvasi M, Hochmuth GJ, Giurcanu MC, George AS, Noel JT, Bartz J, Teplitski M (2013). Factors that affect proliferation of *Salmonella* in tomatoes post-harvest: the roles of seasonal effects, irrigation regime, crop and pathogen genotype. PLoS One.

[46] Guo X, Chen J, Brackett RE, Beuchat LR (2001). Survival of *Salmonellae* on and in tomato plants from the time of inoculation at flowering and early stages of fruit development through fruit ripening. Appl Environ Microbiol.

[47] Montero D, Bodero M, Riveros G, Lapierre L, Gaggero A, Vidal RM, Vidal M (2015). Molecular epidemiology and genetic diversity of *Listeria monocytogenes* isolated from wide variety of ready-to-eat foods and their relationship to clinical strains from listeriosis outbreaks in Chile. Front Microbiol.

[48] Keynan Y, Weber G, Sprecher H (2007). Molecular identification of *Exiguobacterium acetylicum* as the aetiological agent of bacteraemia. J Med Microbiol.

[49] Selvakumar G, Joshi P, Nazim S, Mihra PK, Kundu S, Gupta HS (2009). *Exigubacterium acetylicum* train 1P (MTCC 8707) a novel bacterial antagonist from the North Western Indian Himalayas. World J Microbiol Biotechnol.

[50] Yacoub AT, Katayama M, Tran J, Zadikany R, Kandula M, Greene J (2014). *Bordetella bronchiseptica* in the immunosuppressed population – A case series and review. Mediterr J Hematol Infect Dis.

[51] Sharma NK, Pandey J, Gupta N, Jain RK (2007). Growth and physiological response of *Arthrobacter protophormiae* RKJ100 toward higher concentrations of *o*-nitrobenzoate and *p*-hydroxybenzoate. FEMS Microbiol Lett.

[52] Imirzalioglu C, Hain T, Hossain H, Chakraborty T, Domann E (2010). Erythema caused by localised skin infection with *Arthrobacter mysorens*. BMC Infect Dis.

[53] Duggan JM, Goldstein SJ, Chenoweth CE, Kauffman CA, Bradley SF (1996). *Achromobacter xylosoxidans* bacteremia: report of four cases and review of the literature. Clin Infect Dis.

[54] Townsend SM, Hurrell E, Barron JC, Carrillo CL, Forsythe SJ (2008). Characterization of an extended-spectrum beta-lactamase *Enterobacter hormaechei* nosocomial outbreak, and other *Enterobacter hormaechei* misidentified as Cronobacter (Enterobacter) sakazakii. Microbiology.

[55] Davin-Regli A, Pages JM (2015). *Enterobacter aerogenes* and *Enterobacter cloacae*; versatile bacterial pathogens confronting antibiotic treatment. Front Microbiol.

[56] Lau YY, Yin WF, Chan KG (2014). *Enterobacter asburiae* strain L1: Complete genome and whole genome optical mapping analysis of a quorum sensing bacterium. Sensors.

[57] Broughton WJ, Hern G, Blair M, Beebe S, Gepts P, Vanderleyden J (2003). Beans (*Phaseolus* spp.) – model food legumes. Plant Soil.

[58] Lee JH, Shin H, Park HJ, Ryu S, Han SW (2014). Draft genome sequence of *Xanthomonas axonopodis* pv. Glycines 8ra possessing transcription activator – like affectos used genetic engineering. J Biotechnol.

[59] Surette MA, Sturz AV, Lada RR, Nowak J (2003). Bacterial endophytes in processing carrots (*Daucus carota* L. var. sativus): their localization, population density, biodiversity and their effects on plant growth. Plant Soil.

[60] Xia Y, DeBolt S, Dreyer J, Scott D, Williams MA (2015). Characterization of culturable bacterial endophytes and their capacity to promote plant growth from plants grown using organic or conventional practices. Front Plant Sci.

[61] Lemactud R, Shen SY, Lau M, Fulthorpe R. Bacterial endophytes isolated from plants in natural oil seep soils with chronic hydrocarbon contamination. Front. Microbiol. 2016; https://doi.org/10.3389/fmicb.2016.00755. 10.3389/fmicb.2016.00755 PMC4878295 27252685

