# Supplementary material for: Prevalence of plant beneficial and human pathogenic bacteria isolated from salad vegetables in India
Source: BMC Microbiol. 2017 Mar 14;17:64. doi: 10.1186/s12866-017-0974-x (PMC5348887; doi:10.1186/s12866-017-0974-x)
Supplement: Additional file 1: Table S1. — (a). Morphological and biochemical characterization of bacterial isolates from carrot. (b). Morphological and biochemical characterization of bacterial isolates from cucumber. (c). Morphological and biochemical characterization of bacterial isolates from onion. (d). Morphological and biochemical characterization of bacterial isolates from tomato. (DOC 233 kb) [file 12866_2017_974_MOESM1_ESM.doc]

Additional file 1: Table S1

(a). Morphological and biochemical characterization of bacterial isolates from carrot

| **Bacterial Id** | **Colony morphological characterization** | | **Selective media** | | | | | **Biochemical characterization** | | | | | | | | | | **Bacterial isolate** |
| --- | --- | --- | --- | --- | --- | --- | --- | --- | --- | --- | --- | --- | --- | --- | --- | --- | --- | --- |
|  | **Colour** | **Shape** | **AIA** | **CA** | **EMBA** | **MSA** | **SSA** | **Gram’s staining** | **Bacterial shape** | **Motility** | **I** | **MR** | **VP** | **C** | **Catalase** | **Oxidase** | **Nitrate** |  |
| **SBANHCA1, SBANHCa5, SBANHCa6, SBANHCa13** | **Yellow** | **Circular with regular margin** | **-** | **-** | **-** | **-** | **-** | **-** | **Rod** | **+** | **-** | **+** | **-** | **-** | **+** | **-** | **+** | ***Stenotrophomonas maltophilia*** |
| **SBANHCA2, SBANHCa10, SBANHCa11** | **Pale yellow** | **Circular with regular margin** | **-** | **-** | **-** | **+** | **-** | **+** | **Coccus** | **-** | **-** | **-** | **+** | **-** | **+** | **-** | **+** | ***Staphylococcus aureus*** |
| **SBANHCA3, SBANHCa4** | **Pale grey** | **Circular with irregular margin** | **-** | **-** | **-** | **-** | **+** | **-** | **Rod** | **+** | **-** | **-** | **-** | **+** | **+** | **-** | **+** | ***Salmonella enterica*** |
| **SBANHCA7, SBANHCA9** | **Pale white with Shiny colonies** | **Circular with regular margin** | **-** | **-** | **-** | **-** | **-** | **-** | **Rod** | **+** | **-** | **-** | **+** | **+** | **-** | **+** | **+** | ***Enterobacter aerogenes*** |
| **SBANHCa8** | **Small, greyish white** | **Circular with regular margin** | **-** | **-** | **-** | **-** | **-** | **-** | **Rod** | **+** | **-** | **+** | **+** | **+** | **+** | **+** | **+** | ***Bordetella bronchiseptica*** |
| **SBANHCa12, SBANHCA15, SBANHCa15a, SBANHCA17, SBANHCa17a** | **Pale yellowish** | **Smooth with regular margin** | **-** | **-** | **-** | **-** | **-** | **+** | **Rod** | **+** | **-** | **-** | **+** | **+** | **+** | **+** | **-** | ***Bacillus pumilus*** |
| **SBANHCa1a, SBANHCA14** | **Bluish green** | **Circular with irregular margin** | **-** | **+** | **-** | **-** | **-** | **-** | **Rod** | **+** | **+** | **-** | **+** | **+** | **+** | **+** | **+** | ***Pseudomonas aeruginosa*** |
| **SBANHCA16** | **Pale white** | **Circular , flat with irregular margin** | **-** | **-** | **-** | **-** | **-** | **+** | **Rod** | **+** | **-** | **+** | **-** | **+** | **+** | **-** | **+** | ***Paenibacillus polymyxa*** |
| **SBANHCa14a** | **Yellowish gray** | **Small, circular with regular margin** | **-** | **-** | **-** | **+** | **-** | **+** | **Coccus** | **-** | **-** | **-** | **-** | **-** | **+** | **+** | **+** | ***Staphylococcus sciuri*** |
| **SBANHCa16a** | **Yellowish grey** | **Smooth, flat with regular margin** | **-** | **-** | **-** | **-** | **-** | **+** | **Rod** | **+** | **-** | **+** | **-** | **-** | **+** | **-** | **-** | ***Paenibacillus illinoisensis*** |
| **SBANHCa2a** | **Pale brown** | **Small, circular with regular margin** | **-** | **-** | **-** | **-** | **-** | **-** | **Short rod** | **+** | **-** | **+** | **+** | **+** | **+** | **+** | **+** | ***Enterobacter hormaechei*** |
| **SBANHCa7a** | **Yellowish gray** | **Small, circular with regular margin** | **-** | **-** | **-** | **+** | **-** | **+** | **Coccus** | **-** | **-** | **-** | **-** | **-** | **+** | **+** | **+** | ***Staphylococcus sciuri*** |
| **SBANSCa3** | **Pale white** | **Small, flat with irregular margin** | **-** | **-** | **-** | **-** | **-** | **+** | **Rod** | **+** | **-** | **-** | **+** | **+** | **+** | **+** | ***-*** | ***Bacillus aerophilus*** |
| **SBANSCa4** | **Pale orange** | **Large, flat with irregular margin** | **-** | **-** | **-** | **-** | **-** | **+** | **Rod** | **+** | **-** | **+** | **-** | **-** | **+** | **+** | ***-*** | ***Bacillus cereus*** |
| **SBANSCa5** | **Pale yellow** | **Circular with regular margin** | **-** | **-** | **-** | **-** | **-** | **+** | **Rod** | **-** | **-** | **-** | **-** | **-** | **+** | **-** | ***-*** | ***Microbacterium oleivorans*** |
| **SBANSCa6** | **Pale whitish grey** | **Circular with regular margin** | **-** | **-** | **-** | **-** | **-** | **+** | **Rod** | **+** | **-** | **-** | **-** | **-** | **+** | **+** | ***+*** | ***Arthrobacter nicotianae*** |
| **SBANSCa7** | **Pale white** | **Circular, flat with irregular margin** | **-** | **-** | **-** | **-** | **-** | **+** | **Rod** | **+** | **-** | **-** | **+** | **+** | **+** | **-** | ***+*** | ***Bacillus subtilis*** |
| **SBANSCa10** | **Pale pink** | **Small, circular, flat with irregular margin** | **-** | **-** | **-** | **-** | **-** | **+** | **Rod** | **+** | **-** | **-** | **-** | **+** | **+** | **+** | ***-*** | ***Bacillus flexus*** |

AIA - Aeromonas Isolation agar, CA – Certemaid Agar, EMBA – Eosin Methylene Blue Agar, SSA – Salmonella-Shigella Agar

(b). Morphological and biochemical characterization of bacterial isolates from cucumber

| **Bacterial Id** | **Colony morphological characterization** | | **Selective media** | | | | | **Biochemical characterization** | | | | | | | | | | **Bacterial isolate** |
| --- | --- | --- | --- | --- | --- | --- | --- | --- | --- | --- | --- | --- | --- | --- | --- | --- | --- | --- |
|  | **Colour** | **Shape** | **AIA** | **CA** | **EMBA** | **MSA** | **SSA** | **Gram’s staining** | **Bacterial shape** | **Motility** | **I** | **MR** | **VP** | **C** | **Catalase** | **Oxidase** | **Nitrate** |  |
| **SBANHCu14a, SBANHCu15,**  **SBANHCu15a, SBANHCu16, SBANHCu17, SBANHCu17a, SBANHCu23, SBANHCu23a** | **Pale white** | **Circular with regular margin** | **-** | **-** | **-** | **-** | **-** | **-** | **Short rod** | **+** | **-** | **-** | **+** | **+** | **+** | **+** | **+** | ***Stenotrophomonas rhizophila*** |
| **SBANHCu24** | **Pale yellow** | **Smooth, circular with regular margin** | **-** | **-** | **-** | **-** | **-** | **+** | **Rod** | **-** | **-** | **-** | **-** | **+** | **+** | **-** | **-** | ***Arthrobacter mysorens*** |
| **SBANHCu19, SBANHCu22, SBANHCu25a** | **Oranges yellow** | **Smooth, circular with regular margin** | **-** | **+** | **-** | **-** | **-** | **-** | **Rod** | **+** | **-** | **-** | **-** | **+** | **+** | **+** | **+** | ***Pseudomonas xanthomarina*** |
| **SBANHCu10** | **Pale Yellow** | **Smooth, circular, creamy with regular margin** | **-** | **-** | **-** | **-** | **-** | **+** | **Rod** | **+** | **-** | **+** | **-** | **+** | **+** | **+** | **+** | ***Cellulosimicrobium cellulans*** |
| **SBANHCu12** | **Yellow** | **Circular, moist with regular margin** | **-** | **-** | **-** | **-** | **-** | **+** | **Rod** | **-** | **-** | **-** | **+** | **-** | **+** | **+** | **+** | ***Microbacterium schleiferi*** |
| **SBANHCu14** | **Greyish white** | **Smooth, circular, creamy with regular margin** | **-** | **-** | **-** | **-** | **-** | **-** | **Rod** | **+** | **-** | **-** | **-** | **+** | **+** | **+** | **+** | ***Xanthomonas axonopodis*** |
| **SBANHCu20** | **Brown** | **Rough, circular with irregular margin** | **-** | **+** | **-** | **-** | **-** | **-** | **Rod** | **+** | **-** | **-** | **-** | **+** | **+** | **+** | **+** | ***Pseudomonas stutzeri*** |
| **SBANHCu21** | **white** | **Smooth, circular with regular margin** | **-** | **-** | **-** | **-** | **-** | **-** | **Rod** | **+** | **-** | **-** | **-** | **+** | **+** | **+** | **+** | ***Achromobacter xylosoxidans*** |
| **SBANHCu24a** | **yellow** | **Smooth with regular margin** | **-** | **-** | **-** | **-** | **-** | **+** | **Rod** | **-** | **-** | **+** | **-** | **-** | **+** | **+** | **+** | ***Arthrobacter protophormiae*** |
| **SBANHCu25** | **Brown** | **Smooth , circular with regular margin** | **-** | **+** | **-** | **-** | **-** | **-** | **Short rod** | **+** | **-** | **-** | **-** | **+** | **+** | **+** | **+** | ***Pseudomonas indoloxydans*** |
| **SBANHCu11** | **Creamy white** | **Smooth, circular with regular margin** | **+** | **-** | **-** | **-** | **-** | **-** | **Rod** | **+** | **+** | **+** | **+** | **+** | **+** | **+** | **+** | ***Aeromonas hydrophila*** |
| **SBANSCu4** | **pale white** | **Smooth, circular and slightly irregular margin** | **-** | **-** | **-** | **-** | **-** | **+** | **Rod** | **+** | **-** | **+** | **-** | **-** | **+** | **+** | **+** | ***Geobacillus stearothermophilus*** |
| **SBANSCu7** | **Yellow** | **Small Circular with regular margin** | **-** | **-** | **-** | **+** | **-** | **+** | **Coccus** | **-** | **-** | **-** | **+** | **-** | **+** | **-** | **-** | ***Staphylococcus haemolyticus*** |
| **SBANSCu8** | **white** | **Large, circular with regular margin** | **-** | **-** | **-** | **-** | **-** | **+** | **Rod** | **+** | **-** | **-** | **+** | **+** | **+** | **+** | **+** | ***Bacillus aerius*** |
| **SBANSCu9,**  **SBANSCu11,** | **Creamy white** | **Large, circular with irregular margin** | **-** | **-** | **-** | **-** | **-** | **+** | **Rod** | **+** | **-** | **-** | **-** | **+** | **+** | **-** | **-** | ***Bacillus megaterium*** |
| **SBANSCu10, SBANHCu12a** | **Orange** | **Small, circular with irregular margin** | **-** | **-** | **-** | **-** | **-** | **+** | **Rod** | **+** | **-** | **-** | **+** | **-** | **+** | **-** | **+** | ***Microbacterium arborescens*** |
| **SBANSCu19, SBANSCu22** | **Oranges yellow** | **Smooth, circular with regular margin** | **-** | **+** | **-** | **-** | **-** | **-** | **Rod** | **+** | **-** | **-** | **-** | **+** | **+** | **+** | **+** | ***Pseudomonas xanthomarine*** |
| **SBANSCu20** | **Brownish yellow** | **Smooth, circular with irregular margin** | **-** | **+** | **-** | **-** | **-** | **-** | **Rod** | **+** | **-** | **-** | **-** | **+** | **+** | **+** | **-** | ***Pseudomonas fluorescens*** |
| **SBANSCu21** | **white** | **Smooth, circular with regular margin** | **-** | **-** | **-** | **-** | **-** | **-** | **Rod** | **+** | **-** | **-** | **-** | **+** | **+** | **+** | **+** | ***Achromobacter xylosoxidans*** |

AIA - Aeromonas Isolation agar, CA – Certemaid Agar, EMBA – Eosin Methylene Blue Agar, SSA – Salmonella-Shigella Agar

(c). Morphological and biochemical characterization of bacterial isolates from onion

| **Bacterial Id** | **Colony morphological characterization** | | **Selective media** | | | | | **Biochemical characterization** | | | | | | | | | | **Bacterial isolate** |
| --- | --- | --- | --- | --- | --- | --- | --- | --- | --- | --- | --- | --- | --- | --- | --- | --- | --- | --- |
|  | **Colour** | **Shape** | **AIA** | **CA** | **EMBA** | **MSA** | **SSA** | **Gram’s staining** | **Bacterial shape** | **Motility** | **I** | **MR** | **VP** | **C** | **Catalase** | **Oxidase** | **Nitrate** |  |
| **SBANHO1, SBANHO4, SBANHO12** | **Pale yellow** | **Smooth with regular margin** | **-** | **-** | **-** | **-** | **-** | **+** | **Rod** | **+** | **-** | **-** | **+** | **+** | **+** | **+** | **-** | ***Bacillus pumilus*** |
| **SBANHO2** | **Pale yellow** | **Circular with regular margin** | **-** | **-** | **-** | **+** | **-** | **+** | **Coccus** | **-** | **-** | **-** | **+** | **-** | **+** | **-** | **+** | ***Staphylococcus aureus*** |
| **SBANHO3, SBANHO5, SBANHO8, SBANHO13, SBANHO14** | **Brown** | **Rough, circular with irregular margin** | **-** | **+** | **-** | **-** | **-** | **-** | **Rod** | **+** | **-** | **-** | **-** | **+** | **+** | **+** | **+** | ***Pseudomonas stutzeri*** |
| **SBANHO6** | **Pale yellow** | **Circular , convex with regular margin** | **-** | **-** | **-** | **-** | **-** | **-** | **Rod** | **+** | **-** | **-** | **-** | **-** | **+** | **-** | **-** | ***Xanthomonas fuscans*** |
| **SBANHO7** | **Yellow** | **Circular with regular margin** | **-** | **-** | **-** | **-** | **-** | **-** | **Rod** | **+** | **-** | **+** | **-** | **-** | **+** | **-** | **+** | ***Stenotrophomonas maltophilia*** |
| **SBANHO9** | **Pale greyish** | **Small circular, translucent with regular margin** | **-** | **-** | **-** | **-** | **-** | **+** | **Rod** | **+** | **-** | **+** | **+** | **-** | **+** | **-** | **-** | ***Listeria monocytogenes*** |
| **SBANHO11** | **Greyish white** | **Large, circular with irregular margin** | **-** | **-** | **-** | **-** | **-** | **+** | **Long rod** | **-** | **-** | **-** | **+** | **+** | **+** | **+** | **+** | ***Bacillus anthracis*** |
| **SBANHO15** | **White,** | **Small, circular, concex with regular margin** | **-** | **-** | **-** | **+** | **-** | **+** | **Coccus** | **-** | **-** | **+** | **+** | **-** | **+** | **-** | **+** | ***Staphylococcus epidermidis*** |
| **SBANSO80** | **Orange** | **Small, circular with irregular margin** | **-** | **-** | **-** | **-** | **-** | **+** | **Rod** | **+** | **-** | **-** | **+** | **-** | **+** | **-** | **+** | ***Microbacterium arborescens*** |
| **SBANSO81** | **Pale yellow** | **Smooth with regular margin** | **-** | **-** | **-** | **-** | **-** | **+** | **Rod** | **+** | **-** | **-** | **+** | **+** | **+** | **+** | **-** | ***Bacillus pumilus*** |
| **SBANSO90** | **Pale yellowish grey** | **Small, flat with regular margin** | **-** | **-** | **-** | **+** | **-** | **+** | **Coccus** | **-** | **-** | **-** | **-** | **-** | **+** | **-** | **+** | ***Staphylococcus gallinarum*** |
| **SBANSO54** | **White** | **Large, circular with regular margin** | **-** | **-** | **-** | **-** | **-** | **+** | **Coccus** | **-** | **+** | **+** | **+** | **-** | **-** | **-** | **+** | ***Enterococcus faecium*** |
| **SBANSO82** | **Pale pink** | **Small, circular, flat with irregular margin** | **-** | **-** | **-** | **-** | **-** | **+** | **Rod** | **+** | **-** | **-** | **-** | **+** | **+** | **+** | **-** | ***Bacillus flexus*** |
| **SBANSO83** | **white** | **Circular, with regular margin** | **-** | **-** | **-** | **-** | **-** | **+** | **Rod** | **+** | **-** | **-** | **+** | **+** | **+** | **+** | **+** | ***Bacillus aryabhattai*** |

AIA - Aeromonas Isolation agar, CA – Certemaid Agar, EMBA – Eosin Methylene Blue Agar, SSA – Salmonella-Shigella Agar

(d). Morphological and biochemical characterization of bacterial isolates from tomato

| **Bacterial Id** | **Colony morphological characterization** | | **Selective media** | | | | | **Biochemical characterization** | | | | | | | | | | **Bacterial isolate** |
| --- | --- | --- | --- | --- | --- | --- | --- | --- | --- | --- | --- | --- | --- | --- | --- | --- | --- | --- |
|  | **Colour** | **Shape** | **AIA** | **CA** | **EMBA** | **MSA** | **SSA** | **Gram’s staining** | **Bacterial shape** | **Motility** | **I** | **MR** | **VP** | **C** | **Catalase** | **Oxidase** | **Nitrate** |  |
| **SBANHT1, SBANHT16, SBANHT17** | **Pale white** | **Circular with regular margin** | **-** | **-** | **-** | **-** | **-** | **-** | **Short rod** | **+** | **-** | **-** | **+** | **+** | **+** | **+** | **+** | ***Stenotrophomonas rhizophila*** |
| **SBANHT3** | **Pale pink** | **Small, circular, flat with irregular margin** | **-** | **-** | **-** | **-** | **-** | **+** | **Rod** | **+** | **-** | **-** | **-** | **+** | **+** | **+** | **-** | ***Bacillus flexus*** |
| **SBANHT4** | **Pale white** | **Circular, flat with irregular margin** | **-** | **-** | **-** | **-** | **-** | **+** | **Rod** | **+** | **-** | **-** | **+** | **+** | **+** | **-** | **+** | ***Bacillus subtilis*** |
| **SBANHT7** | **Pale yellowish grey** | **Small, flat with regular margin** | **-** | **-** | **-** | **+** | **-** | **+** | **Coccus** | **-** | **-** | **-** | **-** | **-** | **+** | ***-*** | **+** | ***Staphylococcus gallinarum*** |
| **SBANHT8** | **pale white** | **Smooth, circular with slightly irregular margin** | **-** | **-** | **-** | **-** | **-** | **+** | **Rod** | **+** | **-** | **+** | **-** | **-** | **+** | **+** | **+** | ***Geobacillus stearothermophilus*** |
| **SBANHT9 SBANHT14, SBANHT15** | **Yellowish grey** | **Small, circular with regular margin** | **-** | **-** | **-** | **+** | **-** | **+** | **Coccus** | **-** | **-** | **-** | **-** | **-** | **+** | **+** | **+** | ***Staphylococcus sciuri*** |
| **SBANHT10, SBANHT12** | **pale yellow** | **Smooth with regular margin** | **-** | **-** | **-** | **-** | **-** | **+** | **Rod** | **+** | **-** | **-** | **+** | **+** | **+** | **+** | **-** | ***Bacillus pumilus*** |
| **SBANHT13** | **Yellow** | **Smooth with translucent, regular margin** | **-** | **-** | **-** | **-** | **-** | **+** | **Rod** | **+** | **-** | **+** | **-** | **+** | **+** | **+** | **+** | ***Exiguobacterium acetylium*** |
| **SBANHT19** | **Yellowish orange** | **smooth, glistening, entire with regular margin** | **-** | **-** | **-** | **-** | **-** | **+** | **Rod** | **-** | **-** | **-** | **-** | **+** | **+** | **-** | **+** | ***Microbacterium testaceum*** |
| **SBANHT21** | **Pale Yellow pigmented** | **Smooth, circular, creamy with regular margin** | **-** | **-** | **-** | **-** | **-** | **+** | **Rod** | **+** | **-** | **+** | **-** | **+** | **+** | **+** | **+** | ***Cellulosimicrobium cellulans*** |
| **SBANHT11** | **Pale grey** | **Circular with irregular margin** | **-** | **-** | **-** | **-** | **+** | **-** | **Rod** | **+** | **-** | **-** | **-** | **+** | **+** | **-** | **+** | ***Salmonella enterica*** |
| **SBANST3** | **Pale yellowish** | **Smooth with regular margin** | **-** | **-** | **-** | **-** | **-** | **+** | **Rod** | **+** | **-** | **-** | **+** | **+** | **+** | **+** | **-** | ***Bacillus pumilus*** |
| **SBANST6** | **Pale yellowish** | **Circular, convex with regular margin** | **-** | **-** | **-** | **-** | **-** | **+** | **Rod** | **-** | **-** | **-** | **+** | **-** | **+** | **-** | **-** | ***Terribacillus saccharophilus*** |
| **SBANST7, SBANST11** | **Creamy white** | **Large, circular with irregular margin** | **-** | **-** | **-** | **-** | **-** | **+** | **Rod** | **+** | **-** | **-** | **-** | **+** | **+** | **-** | **-** | ***Bacillus megaterium*** |
| **SBANST8, SBANST12** | **Pale white** | **Circular, flat with irregular margin** | **-** | **-** | **-** | **-** | **-** | **+** | **Rod** | **+** | **-** | **-** | **+** | **+** | **+** | **-** | **+** | ***Bacillus subtilis*** |
| **SBANST6a** | **Creamy pale white** | **Smooth circular with regular margin** | **-** | **-** | **-** | **-** | **-** | **+** | **Rod** | **+** | **+** |  | **+** | **+** | **+** | **+** | **+** | ***Bacillus tequilensis*** |

AIA - Aeromonas Isolation agar, CA – Certemaid Agar, EMBA – Eosin Methylene Blue Agar, SSA – Salmonella-Shigella Agar
